# Genetic Factors Explain a Major Fraction of the 50% Lower Lipoprotein(a) Concentrations in Finns

**DOI:** 10.1161/ATVBAHA.118.310865

**Published:** 2018-03-22

**Authors:** Gertraud Erhart, Claudia Lamina, Terho Lehtimäki, Pedro Marques-Vidal, Mika Kähönen, Peter Vollenweider, Olli T. Raitakari, Gérard Waeber, Barbara Thorand, Konstantin Strauch, Christian Gieger, Thomas Meitinger, Annette Peters, Florian Kronenberg, Stefan Coassin

**Affiliations:** 1 From the Division of Genetic Epidemiology, Department of Medical Genetics, Molecular and Clinical Pharmacology, Medical University of Innsbruck, Austria (G.E., C.L., F.K., S.C.); 2 Department of Clinical Chemistry, Fimlab Laboratories (T.L.); 3 Finnish Cardiovascular Research Center (T.L., M.K.); 4 Department of Clinical Physiology, Tampere University Hospital (M.K.), University of Tampere, Finland; 5 Department of Medicine, Internal Medicine, Lausanne University Hospital, Switzerland (P.M.-V., P.V., G.W.); 6 Department of Clinical Physiology and Nuclear Medicine, Turku University Hospital, Finland (O.T.R.); 7 Research Centre of Applied and Preventive Cardiovascular Medicine, University of Turku, Finland (O.T.R.); 8 Institute of Epidemiology II (B.T., C.G., A.P.); 9 Institute of Genetic Epidemiology (K.S., C.G.); 10 Research Unit of Molecular Epidemiology (C.G.), Helmholtz Zentrum München–German Research Center for Environmental Health, Neuherberg, Germany; 11 German Center for Diabetes Research, Neuherberg, Germany (B.T., A.P.); 12 Institute of Medical Informatics, Biometry, and Epidemiology, Ludwig-Maximilians-Universität, Munich, Germany (K.S.); 13 Institute of Human Genetics, Technische Universität München, Germany (T.M.); 14 Institute of Human Genetics, Helmholtz Zentrum München, Neuherberg, Germany (T.M.); 15 Munich Cluster for Systems Neurology, Germany (T.M.); 16 German Centre for Cardiovascular Research, Partner Site Munich Heart Alliance (A.P.).

**Keywords:** atherosclerosis, genetics, population, lipoprotein(a), risk factors

## Abstract

**Objective—:**

Lp(a) (lipoprotein(a)) concentrations are widely genetically determined by the *LPA* isoforms and show 5-fold interpopulation differences. Two- to 3-fold differences have been reported even within Europe. Finns represent a distinctive population isolate within Europe and have been repeatedly reported to present lower Lp(a) concentrations than Central Europeans. The significance of this finding was unclear for a long time because of the difficult comparability of Lp(a) assays. Recently, a large standardized study in >50 000 individuals from 7 European populations confirmed this observation but could not provide insights into the causes.

**Approach and Results—:**

We investigated Lp(a) concentrations, *LPA* isoforms, and genotypes of established genetic variants affecting Lp(a) concentrations (*LPA* variants, *APOE* isoforms, and *PCSK9* R46L) in the Finnish YFS (Cardiovascular Risk in Young Finns Study) population (n=2281) and 3 Non-Finnish Central European populations (n=10 003). We observed ≈50% lower Lp(a) concentrations in Finns. The isoform distribution was shifted toward longer isoforms, and the percentage of low-molecular-weight isoform carriers was reduced. Most interestingly, however, Lp(a) was reduced in each single-isoform group. In contrast to the known inverse relationship between *LPA* isoforms and Lp(a) concentrations, especially very short isoforms presented unexpectedly low Lp(a) concentrations in Finns. The investigated genetic variants, as well as age, sex, and renal function, explained 71.8% of the observed population differences.

**Conclusions—:**

The population differences in Lp(a) concentrations between Finnish and Central European populations originate not only from a different *LPA* isoform distribution but suggest the existence of novel functional variation in the small-isoform range.

## Abstract 2

Supplemental Digital Content is available in the text.

Lp(a) (lipoprotein(a)) is a major genetically determined cardiovascular risk factor. High Lp(a) plasma concentrations are associated with coronary heart disease, myocardial infarction, aortic valve calcification and stenosis, carotid atherosclerosis, stroke, and venous thromboembolism.^1,2^ Lp(a) concentrations are controlled mostly genetically by the *LPA* locus, which encodes apo(a) (apolipoprotein(a)) as the primary structural protein of Lp(a) and explains 70% to 90% of the Lp(a) concentrations.^1^

The *LPA* gene consists of 10 different KIV (kringle IV) domains (KIV-1 to KIV-10), 1 KV domain (kringle V), and an inactive protease domain.^3^ The KIV-2 domain is encoded by a 5.6-kb large, coding copy number variation, which is present in 1 to >40 repeats^3^ and thus generates >40 isoforms in the population.^3^ The KIV-2 number is inversely correlated to the Lp(a) plasma concentration and explains 30% to 70% of the concentrations.^1^ Low-molecular-weight (LMW) isoforms (≤22 KIV repeats) are associated with ≈4- to 5-fold higher concentrations than high-molecular-weight (HMW) isoforms (>22 KIV).^1^ The smaller allele thus determines the LMW/HMW group assignment.^4^ Although >95% of the population is heterozygous on DNA, only 30% to 70% present both isoforms also in plasma.^3,5^ Null alleles can be caused by large alleles, which tend to be nonexpressed or by 3 known loss-of-function (LOF) mutations.^6–8^

To date, most studies did not identify Lp(a)-modifying genes outside the *LPA* locus. Only recently, 2 additional genes were implicated into Lp(a) metabolism: *APOE*^9–12^ and *PCSK9*.^13,14^ The isoform APOE2 is associated with 15% lower Lp(a) concentrations,^9,10^ whereas the Lp(a)-increasing effect of APOE4^9^ is controversial^9,15^ and vanishes after apo(a) isoform adjustment.^10^ In line with the effects of *PCSK9* inhibitors,^16^ the *PCSK9* LOF mutation R46L (rs11591147) with a minor allele frequency (MAF) of 3% was associated with ≈10% lower Lp(a) concentrations in a Danish population.^13^

Lp(a) concentrations show pronounced interethnical differences. In whites, Malay, Chinese, and Inuit, the distribution of Lp(a) concentration is extremely right skewed^17^ (median Lp(a), ≈10–12 mg/dL), whereas Africans present a rather Gaussian-like distribution and 2- to 3-fold higher Lp(a) levels than whites.^3,5,17^ Indians present intermediary concentrations.^17^ However, lacking standardization of Lp(a) quantification^18^ and the difficult conversion between mass-based (mg/dL) and molar-based (nmol/L) quantification^18^ hampers precise interpopulation comparisons.

The distribution and impact of the apo(a) isoforms differ between ethnicities.^3,5^ The isoform explains 28% of the Lp(a) concentration in Sudanese but 70% in Chinese.^19^ Chinese have a low number of null alleles, whereas Germans, Ghanaians, and San have more 1-isoform carriers, which tend to show lower Lp(a) concentrations than 2-isoform carriers.^20^ In whites, the nonexpressed isoform is mostly the larger allele, whereas in blacks, the distribution of nonexpressed alleles is more U shaped, with an increased proportion of nonexpressed isoforms occurring both among very short and very long isoforms.^5^ Accordingly, differences in the frequency of some Lp(a) concentration-modifying sequence variations (SNPs [single nucleotide polymorphisms] and STRs [short tandem repeats]) have been shown between populations.^3,5^

Within Europeans, Finns represent a genetically distinct population isolate.^21,22^ Lp(a) concentrations have been reported to be lower in Finns than in Central European populations,^7,23–27^ but the significance of this finding was unclear because of the lacking standardization in Lp(a) quantification and small sample numbers in early studies. Only recently, this was confirmed by Waldeyer et al^28^ in a standardized study of 7 European cohorts (n=56 804). The authors confirmed a clear reduction of Lp(a) in Finns and assumed a potential difference in isoform distributions as possible cause.

Indeed, several putative Lp(a)-regulating SNPs present marked differences in MAF in Finns compared with Non-Finnish Central Europeans (NFE). The donor splice-site mutation rs41272114,^7^ which is associated with an Lp(a) reduction by 5.25 mg/dL,^7^ is more frequent in Finns (MAF=0.0635) than in Tyroleans (MAF=0.053)^7^ or in British (PROCARDIS study [Precocious Coronary Artery Disease Study], MAF=0.03^29^; UK Biobank, MAF=0.02).^30^ Also, a second rare splice-site variant (rs143431368)^6^ shows frequency differences between Finns and NFEs (MAF, ≈0.03 in Finns versus 0.003 in NFE) as reported by the Exome Aggregation Consortium (ExAC)^31^ and UK Biobank.^30^ Finally, also differences in the allele frequencies for *PCSK9* R46L and APOE2 have been reported in Finns. Both are associated with reduced Lp(a),^9,10,13^ but, although APOE2 frequency is reduced in Finns by ≈50^32^ and does, therefore, not contribute to the lower Lp(a) concentrations, the *PCSK9* R46L LOF variant is reported to be markedly more frequent (MAF=0.179 versus 0.035 in the ExAC data^31^).

To investigate the factors contributing to the population differences in Lp(a) concentrations, we report here a comprehensive investigation in a Finnish (n=2281) and 3 large Central European studies (n_total_=10 003). By combining SNP and isoform data for all samples, we assess (1) the distribution of isoforms in Finns compared with NFE and (2) dissect the relative contribution of isoforms and established Lp(a)-regulating SNPs to the low Lp(a) levels observed in Finns. Most importantly, all measurements of Lp(a) concentrations and apo(a) isoforms have been done centrally in 1 laboratory to keep methodological influences low.

## Materials and Methods

### Populations

Details on the study populations are reported in Table 1. The CoLaus^33^ study (Cohorte Lausannoise) is a single-center, prospective study, including 6182 randomly selected white subjects aged 35 to 75 years from the city of Lausanne in Switzerland. Only individuals with 4 grandparents of European origin were included in the genetic study. The major aims of the CoLaus study are the investigation of prevalence and determinants of cardiovascular disease and cardiovascular risk factors in the Lausanne population. The analysis data set comprised of 3998 participants with all genotypes and Lp(a) values available.

**Table 1.**
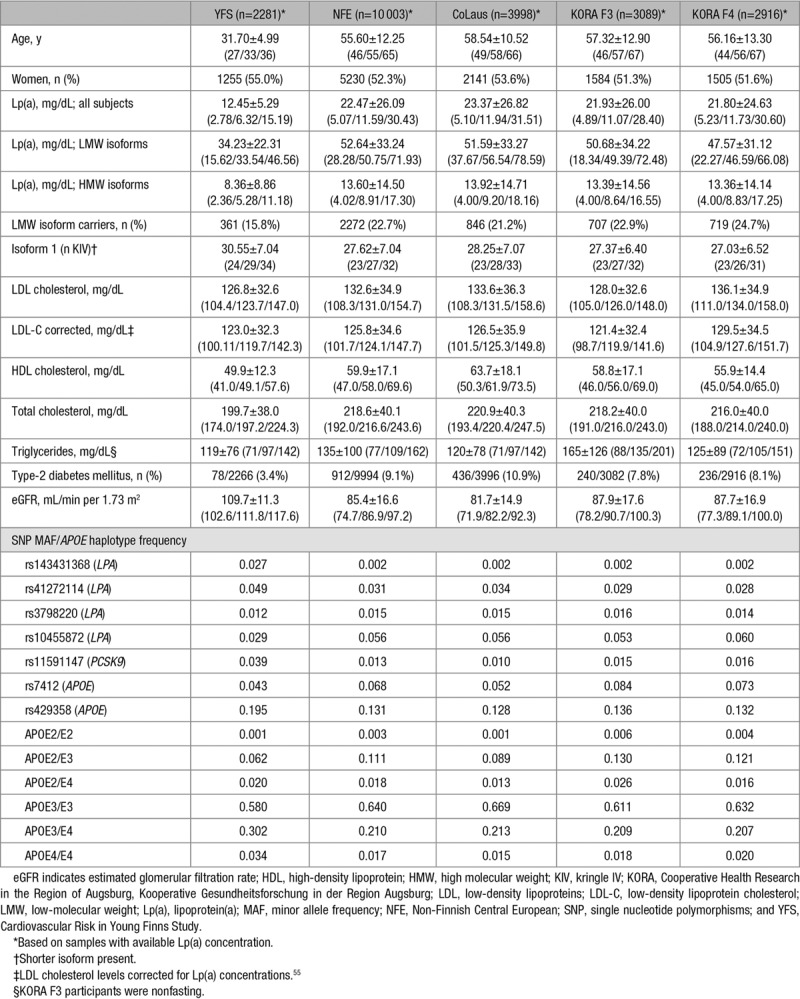
Descriptive Statistics Continuous Variables Shown as Mean±SD and Percentiles (25%/50%/75%)

The KORA cohorts (Cooperative Health Research in the Region of Augsburg, Kooperative Gesundheitsforschung in der Region Augsburg) are several population-based cohorts representative of the general population in Augsburg, Germany, and 2 surrounding counties. Ten-year age–sex strata have been sampled from the 25- to 74-year-old population with a stratum size of 640 subjects. The KORA F3^34^ study was conducted in the years 2004/2005 as a follow-up study of the KORA S3 survey (1994/1995). The KORA F4^34^ study was conducted in the years 2006/2008 and is a follow-up study of the KORA S4 survey (1999/2001). Both studies contain an independent nonoverlapping sample drawn from the same study region. The analyses data set comprised of 3089 participants in KORA F3 and 2916 participants in KORA F4, from which genotypes and Lp(a) values were available.

The YFS (Cardiovascular Risk in Young Finns Study)^35^ is a prospective multicenter study from Finland initiated in 1980 (baseline age, 3–18 years). Children (4320) aged 3, 6, 9, 12, 15, and 18 years were randomly chosen from the population register from the 5 Finnish university cities with medical schools (Helsinki, Kuopio, Oulu, Tampere, and Turku). Several follow-ups during 30 years to investigate childhood risk factors for cardiometabolic outcomes have been performed. Lp(a) phenotype information was available for 2281 participants. The measurements included in this work have been done on materials from the follow-up in the year 2001, where 2283 individuals (63.5% of the original cohort) participated in clinical examinations, and 2620 (72.9%) returned questionnaires.

Informed consent was obtained from each participant, and the studies were approved by the respective institutional review boards.

### Lp(a) Quantification Using ELISA

Lp(a) plasma concentrations were measured using a sandwich ELISA method described by Kronenberg et al^36^ with minor modifications. All measurements were done in the same laboratory (Division of Genetic Epidemiology, Medical University of Innsbruck, Austria).

In brief, the ELISA plates (Nunc-Immuno MicroWell Maxi Sorp flat bottom design, MaxiSorp surface treatment; Thermo Fisher Scientific, Waltham) were coated using an affinity-purified polyclonal rabbit anti-human apo(a) antibody in a final concentration of 5 µg/mL in 1× PBS containing 1 mg/mL NaN_3_. The plates were incubated with 100 µL antibody dilution (3 hours, 37°C), washed 3× (1× PBS+0.05% v/v Tween-20), and blocked with 200-µL 0.1% wt/vol casein in 1× PBS pH 7.3 (30 minutes, 37°C). To ensure measuring each sample within the linear range of optical density, all samples were diluted into the ELISA plate twice (1:150 and 1:1500 in Assay Buffer [Microcoat, Bernried, DE] of 1:30 and 1:1000 predilutions in 1× PBS, pH 7.3). A 7-point standard curve ranging from 0.32 mg/dL to 5 µg/dL was created (with an additional blank representing the zero point). Duplicate determinations of 4 reference samples were used as longitudinal interassay controls.

The coated plates were incubated with the analyte for 1 hour at 37°C. Detection was performed using a horseradish peroxidase-conjugated monoclonal antibody (1A2; in 0.1% wt/vol casein, 1× PBS, pH 7.3) directed against the KIV domain^37^ and not cross-reacting with plasminogen (1 hour, 37°C). After 3 washing steps as described above, 100 µL Blue Star TMB substrate (Adaltis, Guidonia Montecelio, IT) was added (30 minutes, room temperature). Reaction was stopped by adding 50 µL 0.5 mol/L sulfuric acid. Measurement of the absorption (dual wavelength, analyte: 450 nm, reference: 690 nm) was done using a Microplate Reader (BioRad Benchmark Plus; Bio-Rad Laboratories, Hercules), and concentrations were calculated based on the standard curve (expressed as mg/dL). All dilution and pipetting steps were done using liquid handling robotics (Tecan, Männedorf, CH).

### apo(a) Isoform Determination by Western Blot

apo(a) isoform determination has been done as previously described in Kronenberg et al^38^ with minor modifications. All measurements were done in the same laboratory (Division of Genetic Epidemiology, Medical University of Innsbruck, Austria) and evaluated by the same experienced scientist.

In brief, Lp(a) concentrations of each sample were determined by ELISA as described above, and all plasma samples were then diluted with PBS to a standardized Lp(a) concentration of 30 ng/µL. Ten microliters of this dilution were mixed with 20 µL of reducing sample buffer (15.38% v/v glycerol, 7.69% 2-mercaptoethanol, 3.85% v/v SDS, 0.177% v/v 4-ethylmorpholine, and 0.077% wt/vol bromophenol blue) resulting in an Lp(a) concentration of 10 ng/µL. Protein was denatured at 98°C for 5 minutes. Subsequently, 150 ng (15 µL of the 10-ng/µL solution) were applied on a 1.46% agarose gel supplied with 0.08% SDS and separated for 18 hours (0.04 A, constant current). Samples with an Lp(a) concentration <15 ng/µL (ie, 1.5 mg/dL) were not diluted with PBS, but a higher volume of sample was applied on the gel to obtain an equal apo(a) mass. A size standard consisting of a mixture of 5 plasma samples expressing only 1 apo(a) isoform each (13, 19, 23, 27, and 35 KIV repeats, determined by pulsed-field gel electrophoresis^39^) was applied every seventh sample on the gel to detect uneven gel running and minimize distance between samples and standards. After electrophoresis, the proteins were transferred to a polyvinylidenfluorid membrane (Immobilion-P; Millipore, Darmstadt, DE) using semidry blotting (Perfect Blue Semi Dry Blotter; VWR, Vienna, Austria). The membrane was first equilibrated in methanol for 30 seconds, and then rinsed in water and afterward equilibrated in blotting buffer (20% v/v ethanol, 16 mmol/L Tris, and 120 mmol/L glycine). The transfer was done for 45 minutes and 250 mA having the blotting stack semidry in Blotting Buffer. The membrane was then blocked for at least 30 minutes at 37°C using buffer C (85 mmol/L NaCl, 10 mmol/L Tris, 0.2% Triton X-100, and 1% BSA) and incubated in the first antibody (1A2^37^ in a concentration of 168 ng/mL in buffer C) for 2 hours at room temperature on a shaker. After 3 wash steps in TTBS (Tris-buffered saline buffer with Tween-20; 20 mmol/L Tris-HCl pH 7.4, 0.5 mol/L NaCl, and 0.05% Tween-20) of 15 minutes each, the membrane was incubated with horseradish peroxidase-conjugated goat anti-mouse antibody (401253, Millipore, Darmstadt, DE; diluted 1:13 333 in buffer C) for 45 minutes on a shaker at room temperature. Residual secondary antibodies were removed by 3 washing steps (15 minutes in TTBS each); enhanced chemiluminescence substrate (WesternBright Chemiluminescenzce Spray; Biozym, Hessisch Oldendorf, DE) was added, and the signals were detected on an Amersham Hyperfilm for enhanced chemiluminescence (GE Healthcare, Vienna, Austria).

### Genotyping

1000 Genomes^40^-imputed genome-wide genotyping data were available for CoLaus and YFS, whereas genotypes imputed using the haplotype reference consortium^41^ panel were available for KORA F3 and KORA F4. These data were used for evaluation of *LPA* SNPs rs3798220, rs10455872, rs143431368, and rs41272114, as well as *PCSK9* rs11591147 (*PSCK9* R46L) and the *APOE* isoforms E2, E3, and E4 (rs7412 and rs429358). *APOE* haplotypes are defined as follows: APOE2/E2: rs7412=TT and rs429358=TT, APOE2/E3: rs7412=CT and rs429358=TT; APOE2/E4: rs7412=CT and rs429358=CT, APOE3/E3: rs7412=CC and rs429358=TT; APOE3/E4: rs7412=CC and rs429358=CT, APOE4/E4: rs7412=CC and rs429358=CC. Imputation quality is given in Table VII in the online-only Data Supplement and in Mack et al 2017.^10^

### Statistical Methods

In samples heterozygous for apo(a) isoforms, the smaller of the 2 isoforms present is commonly used for assignment to the LMW (≤22 KIV) or the HMW (>22 KIV) groups.^4^ Because genotyping using pulsed-field gel electrophoresis is not feasible on population scale, we refer to the Western blot result throughout the article, when assigning zygosity. For statistical analysis and data presentation of heterozygous samples, the smaller isoform present was termed isoform 1, independent of its size, and the larger isoform was named isoform 2. Accordingly, all isoform-based adjustments and stratifications were done based on the smaller isoform (unless otherwise stated).

Because of the skewed distribution of the Lp(a) trait, nonparametric tests were used to compare median values of Lp(a) between the studies (Mann–Whitney–Wilcoxon test). Dunn post hoc test for multiple comparisons was used to test for significant median differences between all possible pairwise tests between studies. Proportions were tested by χ2 test. In regression and mediation models, inverse-normal transformation was used for Lp(a) to adhere to the normal distribution assumption for statistical tests. Equal distribution of isoforms within carriers of rs10455872 and rs3798220 in YFS and NFE was tested using the Kolmogorov–Smirnov test.

We used a multivariable regression model to evaluate the joint influence of the SNPs rs10455872, rs3798220, rs143411368, rs41272114, and rs11591147, APOE2 and APOE4-carriers, the isoforms (isoform 1), age, sex, and estimated glomerular filtration rate (eGFR) on Lp(a). Imputed genotype scores were used on a continuous scale, which corresponds to an additive coding. The eGFR was included to account for renal function, which is one of the few established nongenetic modifiers of Lp(a) concentrations.^42,43^ eGFR was calculated according to Levey et al,^44^ (CDK EPI [Chronic Kidney Disease Epidemiology Collaboration] creatinine equation). For the isoforms, a nonlinear spline was used. The multivariable regression models were performed in each of the 4 populations individually and for all Non-Finnish European studies combined (NFE) using a mixed model accounting for the study as a random effect (package mgcv in R).

To investigate whether the lower Lp(a) values in Finns can be explained by the evaluated variables, we performed a formal mediation analysis. This means that the effect on Lp(a) of being Finnish is decomposed into the effect portions, which are mediated by the isoforms, by the SNPs, and by other factors, which differ between YFS and the Non-Finnish European studies (see the Results section). Because the participants in the YFS are younger than the participants in the other Non-Finnish studies, age and, in consequence, better kidney function could also possibly explain the difference in Lp(a) distribution. Therefore, eGFR and age were also included as potential mediating variables. Technically, a binary variable (Finnish versus Non-Finnish) was created and regressed onto inverse-normal transformed Lp(a) values. This effect is denoted as the total effect. Then, this simple regression model was adjusted for the potential mediating variables (age, sex, eGFR, isoform 1, and the investigated SNPs). The percentage by which the total effect is attenuated is the relative mediated effect, which can be decomposed into their single parts (indirect effects, herein after referred to as explained effects). The remaining effect is the direct effect of the variable being Finnish. This is actually the remainder, which cannot be explained by the included variables (herein after referred to as unexplained effects). Assumptions of the mediation model are (1) association of the mediators with the outcome (this was set to a *P* value <0.1) and (2) association of the exposure (the Finnish versus Non-Finnish effect) with the outcome (*P* value, <0.1). Only those variables were included in the mediation analysis that fulfilled these assumptions. Model assumptions, decomposition into single indirect effects (time of resampling, n=100), and bootstrap confidence intervals (number of times of bootstrap resampling, n=500) were performed using the package mma in R. All statistical analyses were done with R (http://www.r-project.org).

## Results

### YFS Present Reduced Lp(a) Concentrations Compared With Central Europeans

**Figure 1.**
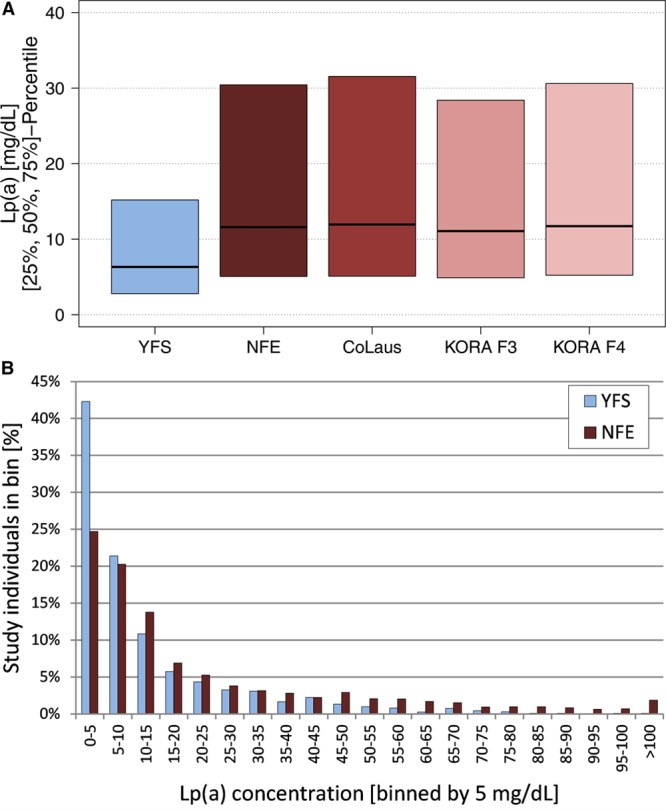
Distribution of Lp(a) (lipoprotein(a)) concentrations. **A**, Median Lp(a) concentrations for each population and the combined Non-Finnish Europeans (NFE) group. YFS (Cardiovascular Risk in Young Finns Study) shows ≈50% lower Lp(a) concentrations. Because of the large variance of Lp(a) concentrations, only medians and 25%/75% percentiles are shown to enhance comprehensibility. Full figure is given in Figure I in the online-only Data Supplement. **B**, Frequencies of Lp(a) concentrations for YFS and NFE stratified by 5 mg/dL strata. The 0 to 5 mg/dL group is markedly larger, and the relative frequencies in almost all strata >30 mg/dL are substantially smaller in YFS than in NFE. Figure II in the online-only Data Supplement shows nonclustered NFE. KORA, Cooperative Health Research in the Region of Augsburg, Kooperative Gesundheitsforschung in der Region Augsburg.

We determined Lp(a) concentrations and apo(a) isoforms in the YFS (n=2281) and in the 3 Central European population CoLaus (n=3998), KORA F3 (n=3089), and KORA F4 (n=2916). All measurements were performed in the same laboratory using standardized ELISA and Western blot assays. Median Lp(a) concentrations were similar in all Central European populations (*P*>0.59 for all pairwise comparisons between Central European populations) but significantly different between YFS and all Central European studies (Figure 1A; Table I in the online-only Data Supplement; all *P* ≤1.38×10^−58^ for all pairwise comparisons using Dunn post hoc test). Therefore, for all subsequent analyses, CoLaus, KORA F3, and KORA F4 were combined to the Non-Finnish Europeans (NFE) population. The median Lp(a) concentration in YFS was significantly lower than in NFE (6.3 versus 11.6 mg/dL; *P*=8.15×10^−91^; Figure 1A). This difference was present both in men and women and when restricting the analysis only to the age range ≥32 to ≤39 years allowing a similar age distribution of all studies (Table I in the online-only Data Supplement). When stratifying Lp(a) concentrations into 5-mg/dL strata, concentrations of 0 to 5 mg/dL were overrepresented in YFS compared with NFE (42.3% versus 24.7%; *P*=5.22×10^−64^; Figure 1B). Accordingly, less individuals showed concentrations >30 mg/dL and >50 mg/dL, which are commonly deemed as thresholds for increased cardiovascular risk^45,46^ (>30 mg/dL, 12.1% versus 25.3%; >50 mg/dL, 3.9% versus 14.1%; *P*=2.32×10^−41^ and *P*=1.17×10^−41^; Figure III in the online-only Data Supplement).

### Differences in Isoform Distributions Between YFS and NFE

The Lp(a) concentration is highly dependent on the number of KIV repeats. We, therefore, investigated whether the apo(a) isoform distribution is shifted toward larger isoforms in YFS compared with NFE, which could potentially explain the lower Lp(a) levels observed in YFS.

**Figure 2.**
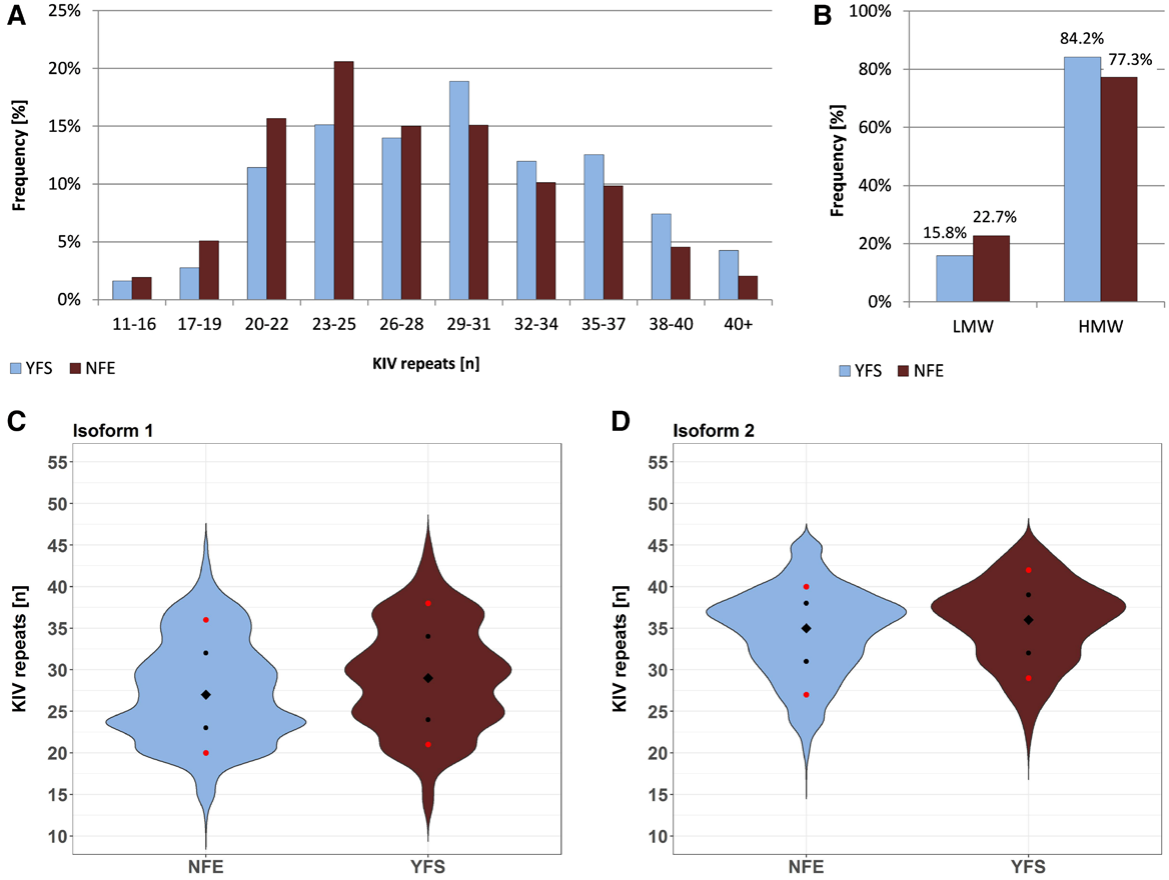
apo(a) (apolipoprotein(a)) isoform distribution in YFS (Cardiovascular Risk in Young Finns Study) and Non-Finnish Central Europeans (NFE). **A**, Isoform 1 in YFS and NFE stratified by groups of 3 KIV (kringle IV) repeats (except the group with shortest isoforms 11–16). YFS shows a higher frequency of carriers >28 KIV repeats, compared with NFE. The number of carriers in each isoform stratum is given in Table IV in the online-only Data Supplement. **B**, Low-molecular-weight (LMW; at least 1 isoform with ≤22 KIV repeats) and high-molecular-weight (HMW) carriers (only isoforms with >22 repeats) in YFS and NFE. HMW individuals are more common in YFS. *P*=5.83×10^−13^. **C** and **D**, apo(a) isoform distribution of the 2 alleles. The violin plots show distributions of the smaller allele (isoform 1) and the larger allele (isoform 2, if detectable). The isoform distribution in YFS is shifted toward larger alleles. Diamonds, median; black dots, 25th and 75th percentiles; red dots, 10th and 90th percentiles. For details and unclustered NFE data, see Figure IV in the online-only Data Supplement and Table III in the online-only Data Supplement.

Isoforms ≤25 KIV repeats were more frequent in NFE than in YFS, whereas YFS presented higher frequencies of isoforms with >28 KIV domains (Figure 2A). Accordingly, LMW frequency was significantly reduced in YFS compared with the NFE populations (361/2282 [15.8%] versus 2272/10 003 [22.7%]; *P*=5.83×10^−13^; Figure 2B). The median size of the smaller isoform (isoform 1) was larger in YFS than in NFE (29 versus 27 KIV repeats; *P*=8.21×10^−37^; Figure 2C; Table II in the online-only Data Supplement). In heterozygous individuals, the differences in the distribution of the larger isoform (isoform 2) were less pronounced but still significant (36 versus 35 KIV repeats; *P*=3.54×10^−12^; Figure 2D; Table II in the online-only Data Supplement).

**Figure 3.**
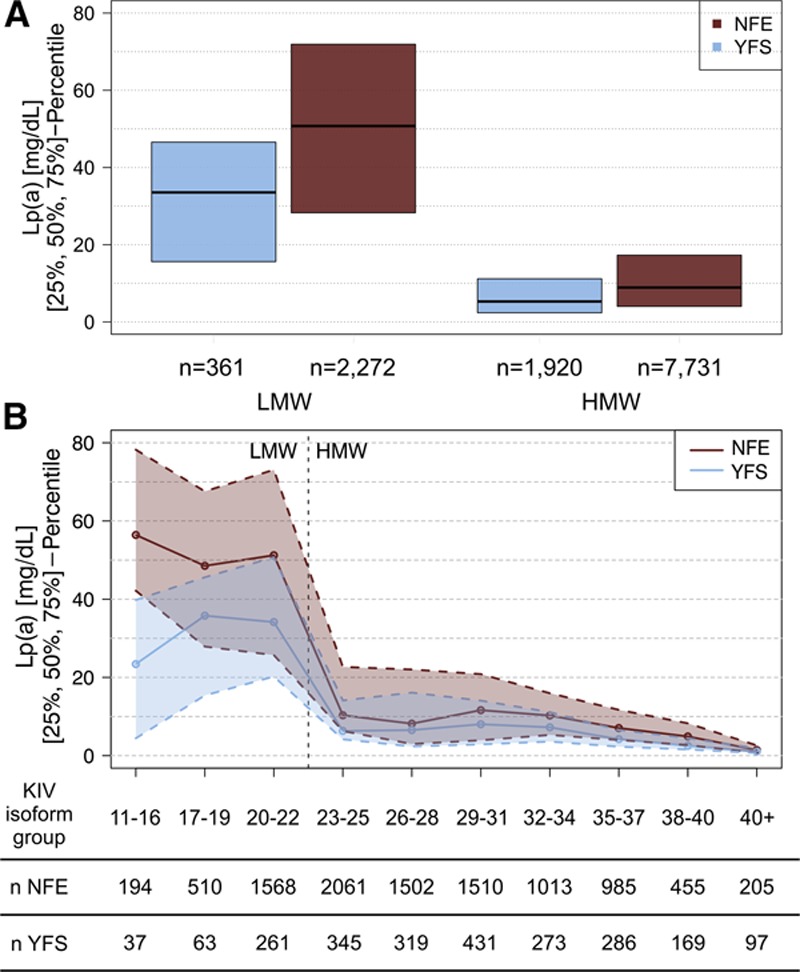
Lp(a) (lipoprotein(a)) concentrations stratified by apo(a) (apolipoprotein(a)) isoform size. **A**, Median Lp(a) concentration stratified by low-molecular-weight (LMW)/high-molecular-weight (HMW) classification. In both groups, YFS (Cardiovascular Risk in Young Finns Study) presents lower median Lp(a) concentrations than Non-Finnish Central Europeans (NFE). The nonclustered populations are shown in Figure V in the online-only Data Supplement. **B**, Median Lp(a) concentrations stratified by isoform size in groups of 3 to 5 KIV (kringle IV) isoforms (based on isoform 1). YFS individuals express lower Lp(a) concentrations than NFE individuals in all isoform groups. In the group 11 to 16 KIV repeats, Finns have an even lower Lp(a) concentration than in the larger 17 to 19 KIV repeats group. Stratified number of carriers for each population and median Lp(a) concentrations are given in Tables IV and V in the online-only Data Supplement and Figure VI in the online-only Data Supplement.

Interestingly, the Lp(a) concentrations were lower in YFS than in NFE even within the LMW and HMW groups (Figure 3A). Because this could arise from a shift of the isoform distribution toward higher isoforms within the groups (thus reducing the group mean without changing group assignment), we stratified the Lp(a) isoforms into more granular subgroups as done in earlier studies.^47^ YFS showed reduced Lp(a) concentrations in all subgroups (Figure 3B; Table IV in the online-only Data Supplement). Also, the percentage of homozygous null allele carriers (ie, samples that do not present any of the 2 *LPA* isoforms in plasma as detectable by Western blotting) was higher in YFS than in NFE (n=36 [1.6%] versus n=70 [0.7%]; *P*=7.25×10^−5^). However, the median Lp(a) concentration of the entire YFS cohort increased only marginally from 6.32 to 6.46 mg/dL after exclusion of the homozygous null allele carriers.

### Frequency of Established Lp(a)-Regulating SNPs

The *APOE* isoforms and PCSK9 R46L have been recently established as novel genetic regulators of Lp(a).^9,10,13^ As described in detail in the Introduction section, both genetic variants show differences in allele frequencies between Finns and Central Europeans. The same applies to 2 well-known *LPA* splice-site variants^6,7^ (Table 1). We, therefore, evaluated to which extent these variants contribute to the differences in Lp(a) concentrations between YFS and NFE. YFS presented a higher frequency of E3/E4 and E4/E4 (*P*=3.72×10^−20^ and *P*=1.04×10^−6^, respectively) and lower frequencies of E2/E3 (*P*=1.41×10^−11^; Table 1). Conversely, rs11591147, rs143431368, and rs41272114 were more frequent in YFS than in NFE (Table 1; *P*=4.79×10^−29^, *P*=6.77×10^−83^, and *P*=1.44×10^−8^, respectively). They represent, therefore, valid putative determinants for the observed differences in Lp(a).

Moreover, the *LPA* variants rs3798220^48^ and rs10455872^48^, which have been reported to tag small isoforms (19–21 and 17–20 KIV repeats, respectively),^48^ were included. Both showed the same isoform distribution in YFS as in NFE (*P*=0.61 and *P*=0.20; Figure VII in the online-only Data Supplement). The rs10455872 frequency was ≈50% lower in YFS (which is also in line with the lower frequency of short isoforms), whereas no pronounced frequency difference was observed for the rarer rs3798220 variant.

### Association of Potential Mediating Factors With Lp(a) Concentrations

**Figure 4.**
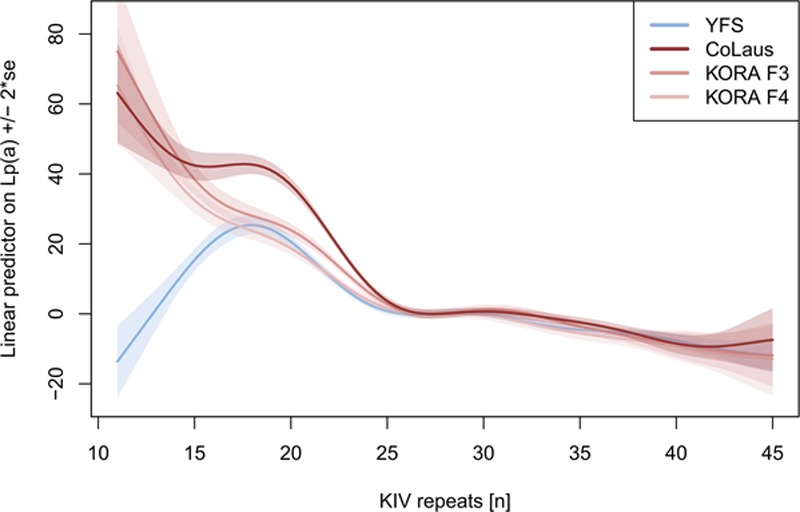
Nonlinear spline, showing the effect of isoform 1 (ie, in heterozygotes, the smaller allele present) on untransformed Lp(a) (lipoprotein(a)) concentrations. The splines are centered at 0 at isoform 1=27 (median on the Non-Finnish Central Europeans studies). The splines are derived from the multiple regression model additionally adjusting for the variables age, sex, estimated glomerular filtration rate, rs143411368, rs41272114, rs11591147, *APOE2*, and *APOE4*-carriers (Table V in the online-only Data Supplement). The spline for inverse-transformed Lp(a) is shown in Figure VIII in the online-only Data Supplement. YFS, Cardiovascular Risk in Young Finns Study; KORA, Cooperative Health Research in the Region of Augsburg, Kooperative Gesundheitsforschung in der Region Augsburg; KIV, kringle IV.

We investigated *LPA* isoform distribution, *LPA* and *PCSK9* LOF frequencies, and *APOE* isoform frequencies, as well as sex, age, and renal function, estimated by eGFR^1,5^ as potential mediators of the observed population differences. Results from multiple regression models for each factor on Lp(a) are reported in Table 2 (YFS and NFE) and Table V in the online-only Data Supplement (single studies). Among the genetic factors, isoforms were highly associated with Lp(a) in a nonlinear fashion (Figure 4), whereas *APOE2* and the SNPs rs143411368 and rs41272114, which are more frequent in the YFS, showed an Lp(a)-decreasing effect. Conversely, rs10455872 and rs378220 were associated with increased Lp(a), but only rs10455872 shows a pronounced frequency difference between YFS and NFE and thus potentially contributes to the Lp(a) difference. No association was observed for *PCSK9* R46L (rs11591147) and *APOE4* carrier status, despite their frequency difference between YFS and NFE.

**Table 2.**
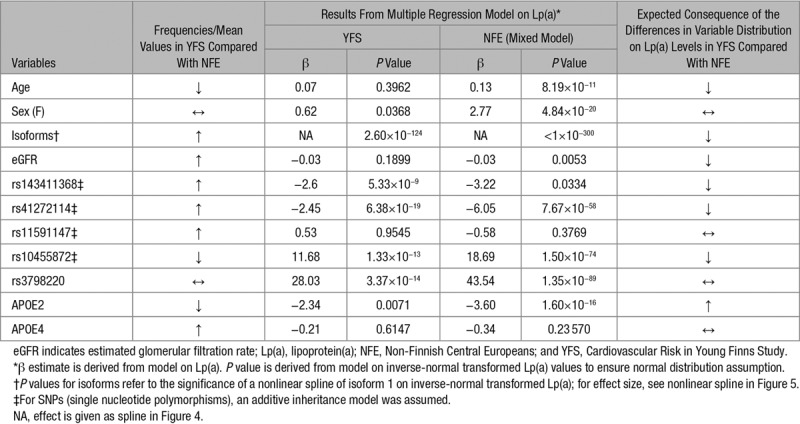
Results From Multiple Regression Analyses in the YFS and in NFE Combined (Using a Random Effects Model)

The multiple adjusted effect of isoform 1 on Lp(a) is shown in Figure 4. In general, Lp(a) decreases with increasing KIV repeats, although not linearly. Because in YFS the distribution of isoforms is shifted to higher repeat numbers, lower Lp(a) values are expected. Both in YFS and NFE, Lp(a) decreases rather sharply at >22 repeats. The nonlinear relationship is consistent in all 3 Non-Finnish studies and is also consistent for HMW isoforms in YFS. Interestingly, the effect of isoform 1 being <20 KIV repeats on Lp(a) concentrations differs between YFS and NFE. Although in NFE the Lp(a) concentrations still rise for isoforms <20, the Lp(a) values decrease in YFS.

Among the study-specific factors, age and female sex were associated with increased Lp(a) values, whereas eGFR, which is higher in the YFS, was associated with a decreased Lp(a) (although not significantly). Because YFS individuals are younger and present better renal function than those in the NFE studies, Lp(a) values are lower in YFS also in consequence of the age effect.

### Mediation Analysis Explaining the Differences in Lp(a) Concentrations Between Finns and Non-Finns

**Figure 5.**
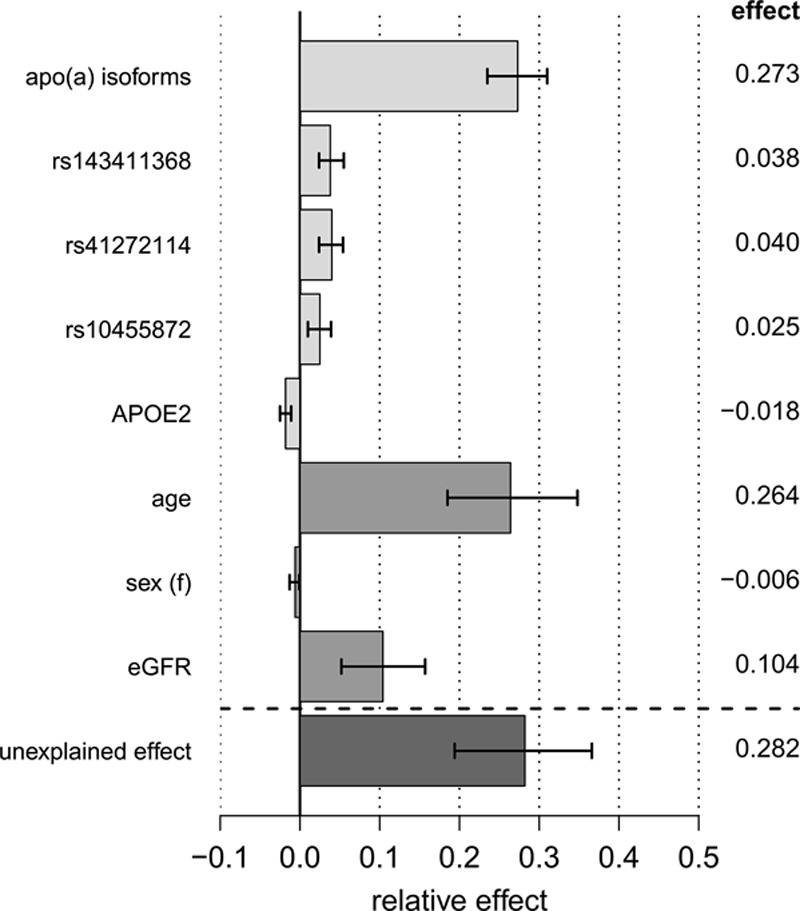
Mediation analysis explaining the differences in Lp(a) (lipoprotein(a)) concentrations between YFS (Cardiovascular Risk in Young Finns Study) and Non-Finnish Central Europeans: decomposition of the total effect on inverse-normal transformed Lp(a) values into explained and unexplained relative effects (including 95% confidence interval). Exact values are reported in Table VII in the online-only Data Supplement. eGFR, estimated glomerular filtration rate; apo(a), apolipoprotein(a).

The multiple regression model (Table 2) indicates that both genetic and environmental factors, which are associated with reduced Lp(a) and which are more frequent in YFS than in NFE, contribute to lowering Lp(a) in YFS. To identify the relative contribution of these factors, we conducted a formal mediation analysis. All variables of the multiple regression models were included as potential mediators of the “Finns effect” on Lp(a), except SNP rs11591147, which was not significant in the multiple regression model and thus did not fulfill the criteria to be included as a mediator (Table VI in the online-only Data Supplement), and rs3798220, which was not identified as an independent modifier (Table VII in the online-only Data Supplement; column *P* value 2). Altogether, 71.8% of the Finns effect is determined by the included mediators, which leaves 28.2% unexplained (Figure 5). The largest contributors to reduced Lp(a) concentrations in YFS are the isoforms (27.3%), followed by age (26.4%), eGFR (10.4%), rs41272114 (4.0%), and rs143411368 (3.8%), whereas *APOE2* frequency contributed inversely (−1.8%).

## Discussion

Lp(a) concentrations show pronounced interethnic and interpopulation variability.^5,17,19^ Several reports showed lower Lp(a) concentrations in Finns than in other European populations,^23–27,49^ but the small sample numbers of early studies and the nonstandardized Lp(a) quantification methods hampered a direct comparison. Waldeyer et al^28^ recently confirmed lower median Lp(a) concentrations (≈−58%) in the Finnish FINRISK cohort compared with Central European populations. However, the causes could not be addressed. Various causes are conceivable: (1) differences in the isoform distribution, (2) differences in the frequency of Lp(a)-lowering variants, or (3) differences in modifier genes.

To dissect the relative contribution of these factors in a standardized study, we determined apo(a) isoforms and Lp(a) concentrations with the same assays for 10 003 Central European whites from 3 population-based studies (NFE) and 1 Finnish study (YFS; n=2281).^10^ The contributions of isoform distribution, *LPA* LOF mutations and variants, *APOE* genotypes, and *PCSK9* R46L were assessed.

We found that a consistent part of the differences in Lp(a) concentrations can be explained by the common action of (1) isoform distribution, (2) *LPA* variants, and (3) *APOE* genotypes. Additionally, we observed that in Finns, the Lp(a) concentrations were reduced in all apo(a) isoform groups.

The isoform distribution in Finns was shifted toward longer isoforms (Figure 2), which accounted for 27% of the interpopulation differences in Lp(a) concentrations (Figure 5). It thus represents the largest contributor to the interpopulation differences without being, however, the only causal factor. This is in line with previous reports showing that differences in isoform distribution among populations only partially account for interpopulation differences in Lp(a) concentrations.^1,5^

We found that in YFS, the Lp(a) concentrations were reduced even within the same-isoform group. This mirrors the differences between blacks and whites, where blacks present higher Lp(a) concentrations over the whole range of isoforms.^17,50^The effect was most striking in the 11 to 16 KIV repeats group, which showed even lower Lp(a) than the 17 to 19 KIV repeats group (Figures 3B and 4). Finns have a higher frequency of LOF mutations and variants in conserved noncoding regions than Central Europeans.^6,51^ Because *LPA* is reported to be LOF tolerant by ExAC (according to the pLI/pRec scores on the ExAC website^31^) and by phenome-wide studies,^30^ and it has been shown that Lp(a)-modifying SNPs can cluster with particular isoforms^3,52^; it is tempting to speculate that novel functional variants may exist in the 11 to 16 KIV repeat carriers in YFS. Indeed, especially short isoforms according to the Western blot might represent truncated protein forms caused by novel nonsense or splice-site variants. Such truncated forms have been observed previously in Tyroleans with low Lp(a).^8^

Several studies proposed that (even frequent) SNPs contribute to interethnic differences in Lp(a) levels.^53,54^ The *APOE* isoforms vary between Finns and Central Europeans, with *APOE2* being rarer and *APOE4* being more frequent in Finns. Although the individual impact of these genetic variants on Lp(a) has been described previously,^6,7,9,10,12^ their cumulative contribution in establishing low Lp(a) levels in Finns has not been evaluated to date. We showed that the frequency distribution of 2 *LPA* LOF variants and rs10455872 contributed for a total of ≈10% to the population difference. Conversely, *APOE2* counteracts this by increasing Lp(a) and so contributes to closing the gap between the populations (Figure 5; Table VI in the online-only Data Supplement). Among the *APOE* genotypes, only *APOE2* showed an appreciable contribution in the mediation analysis in this sample set, which is in line with previous results^10^ (Figure 5; Table VI in the online-only Data Supplement). Of note, rs41272114—a major contributor in our mediation analysis—has been proposed also as a cause for the Lp(a) differences between whites and Africans.^54^ No effect of rs11591147 was found, which is likely because of its small absolute effects on Lp(a) concentrations (1 mg/dL).^13^ We could also not confirm the high MAF (17.9%) of rs11591147 reported in Finns by ExAC (Table 1), but we rather support the MAF determined in the SISu (Sequencing Initiative in Suomi) project (4.2%).^6^

Importantly, the focus of the work at hands was to show that the observed differences in Lp(a) are not just because of a shifted isoform distribution and evaluate the contribution of known functional factors affecting Lp(a) concentrations. This left ≈28% of variance unexplained and suggests the existence of additional functional variation. Various types of genetic variants may contribute to this observation. The KIV-2 region plays a special role because it encompasses a considerable part of the *LPA* gene but is currently not captured by common genetic data set. However, also differences in the effect magnitude or frequency of known variation outside the KIV-2 may play role. Comparative ultradeep sequencing studies and a dedicated study design will be required to pinpoint the genetic determinants of the population differences reported here.

### Strengths and Limitations

Our study presents strengths and limitations. To the best of our knowledge, here we present the largest investigation of Lp(a) concentrations in European populations accounting for Lp(a) isoform data to date. All measurements and Western blots were done in a single laboratory using the same methods in all populations. The YFS individuals are younger and present better renal function—a major determinant of Lp(a) levels^1^—than the NFE. Accordingly, environmental differences account for 34% of the population differences, and age and eGFR were the second largest contributors to the population differences. This underscores the importance of matching these factors, despite the often alleged robustness Lp(a) to nongenetic influences.

The pronounced effect of age is surprising and could reflect kidney function parameters, which are not reflected by eGFR, other unmeasured age-related confounders (eg, hormonal status), or undetermined study-specific confounders, which are specific for the YFS study. The difference between YFS and NFE is present in both sexes (Table I in the online-only Data Supplement) and also when looking only at the age range ≥32 to ≤39, where all studies overlap. This indicates that the observed Lp(a) reduction is not only because of different proportion of postmenopausal individuals in NFE. Finally, it has to be noted that the mediation analysis assumes a linear and homogeneous effect of the factors. Therefore, the differing effect of low isoforms on Lp(a) in YFS cannot be fully accounted for and may contribute to the unexplained effect.

Importantly, no other study with combined information on Lp(a) concentrations and isoform distribution in Finns was available until now, except 1 small study in 181 individuals.^27^ We could successfully identify and quantify determinants of the Lp(a) differences between Finns and NFE and show that a limited number of genetic elements (isoforms and SNPs) already explain remarkable ≈37% of the population difference. This may indicate that a small number of factors can already explain a consistent part of differences of Lp(a) between Finns and NFE populations. Finally, the availability of Western blot data allowed identifying a surprising Lp(a)-lowering effect in short isoforms in YFS, which warrants further research.

### Conclusion

We demonstrate that the lower Lp(a) concentrations in Finns are not caused solely by differences in isoform distribution but are lowered across the whole range of isoforms. Frequency differences of common LOF mutations and *APOE* isoforms significantly contribute to these findings but do not fully explain it. Of particular note, especially very small isoforms with 11 to 16 KIV repeats present massively lowered Lp(a) concentrations, which might hint toward the existence of novel LOF mutations. Therefore, future sequencing studies in *LPA* might be fruitful to identify novel Lp(a)-lowering variants.

## Acknowledgments

This work was part of the bachelor thesis in Biomedical Sciences of G. Erhart at the Health University of Applied Sciences Tyrol.

## Sources of Funding

This study was supported by the Austrian Science Fund (FWF) Project Number P266600-B13 to C. Lamina. Other funding sources are given in the online-only Data Supplement.

## Disclosures

None.

## Supplementary Material

## Notes

Highlights

- Lp(a) (lipoprotein(a)) concentrations are massively reduced by ≈50% in Finns (n=2281) compared with Central Europeans (n=10 003).
- Finns presented a shift in apo(a) isoform distribution toward larger isoforms and frequency differences in established Lp(a)-regulating genetic variants (*LPA* loss-of-functions, *APOE*, and *PCSK9*).
- Lp(a) was reduced over the whole isoform range. Of particular note, very small isoforms showed surprisingly low Lp(a) concentrations.
- 71.8% of the differences between the populations can be explained by a combination of genetic and environmental factors.
