# Supplementary material for: Genetic Factors Explain a Major Fraction of the 50% Lower Lipoprotein(a) Concentrations in Finns
Source: Arterioscler Thromb Vasc Biol. 2018 Mar 22;38(5):1230–41. doi: 10.1161/ATVBAHA.118.310865 (PMC5943067; doi:10.1161/ATVBAHA.118.310865)
Supplement: Supplementary file 1 [file atv-38-1230-s001.pdf]

Major Resources Table

Antibodies

Target antigen	Vendor or Source	Catalog #	Working concentration	Lot # (preferred but not required)
affinity-purified polyclonal rabbit anti-human apo(a) antibody	produced in-house	produced in-house	5 µg/ml in 1x PBS containing 1 mg/mL NaN_3 for ELISA	- -
horseradish-peroxidase-conjugated monoclonal anti-apo(a) antibody 1A2	produced in-house	produced in-house	Conjugate stock stored 1:7,000 in glycerol, then diluted 1:4,800 in Assay Buffer (Microcoat, Bernried, D)	-
1A2 monoclonal anti-apo(a) antibody for Western blotting	produced in-house	produced in-house	168 ng/ml in Buffer C (85 mM NaCl, 10 mM TRIS, 0.2% Triton X-100, 1% BSA)	-
horseradish-peroxidase conjugated goat anti-mouse	Millipore	401253	1:13,333 in Buffer C (85 mM NaCl, 10 mM TRIS, 0.2% Triton X-100, 1% BSA)	-
