# Supplementary material for: Genetic Factors Explain a Major Fraction of the 50% Lower Lipoprotein(a) Concentrations in Finns
Source: Arterioscler Thromb Vasc Biol. 2018 Mar 22;38(5):1230–41. doi: 10.1161/ATVBAHA.118.310865 (PMC5943067; doi:10.1161/ATVBAHA.118.310865)
Supplement: Supplementary file 2 [file atv-38-1230-s002.pdf]

SUPPLEMENTAL MATERIAL

Genetic factors explain a major fraction of the 50% lower lipoprotein(a) concentrations in Finns

Running title: Genetic factors lower Lp(a) in Finns

Gertraud Erhart[1], Claudia Lamina[1], Terho Lehtimäki[2], Pedro Marques-Vidal[3], Mika Kähönen[4], Peter Vollenweider[3], Olli T Raitakari[5,6], Gérard Waeber[3], Barbara Thorand[7,8], Konstantin Strauch[9,10], Christian Gieger[7,9,11], Thomas Meitinger[12,13,14], Annette Peters[7,8,15], Florian Kronenberg[1], Stefan Coassin[1]

[1] Division of Genetic Epidemiology, Department of Medical Genetics, Molecular and Clinical Pharmacology, Medical University of Innsbruck, Innsbruck, Austria,
[2] Department of Clinical Chemistry, Fimlab Laboratories, and Finnish Cardiovascular Research Center - Tampere, Faculty of Medicine and Life Sciences, University of Tampere, Tampere 33520, Finland
[3] Internal Medicine, Lausanne University Hospital, 1015 Lausanne, Switzerland
[4]Department of Clinical Physiology, Tampere University Hospital, and Finnish Cardiovascular Research Center - Tampere, Faculty of Medicine and Life Sciences, University of Tampere, Tampere 33521, Finland
[5] Department of Clinical Physiology and Nuclear Medicine, Turku University Hospital, Turku, Finland
[6] Research Centre of Applied and Preventive Cardiovascular Medicine, University of Turku, Turku, Finland
[7] Institute of Epidemiology II, Helmholtz Zentrum München—German Research Center for Environmental Health, 85764 Neuherberg, Germany
[8] German Center for Diabetes Research (DZD), 85764 Neuherberg, Germany
[9] Institute of Genetic Epidemiology, Helmholtz Zentrum München—German Research Center for Environmental Health, 85764 Neuherberg, Germany
[10] Institute of Medical Informatics, Biometry and Epidemiology, Chair of Genetic Epidemiology, Ludwig-Maximilians-Universität, 81377 Munich, Germany
[11] Research Unit of Molecular Epidemiology, Helmholtz Zentrum München—German Research Center for Environmental Health, 85764 Neuherberg, Germany
[12] Institute of Human Genetics, Technische Universität München, 81675 München, Germany
[13] Institute of Human Genetics, Helmholtz Zentrum München, 85764 Neuherberg, Germany
[14] Munich Cluster for Systems Neurology (SyNergy), 81377 Munich, Germany
[15] DZHK (German Centre for Cardiovascular Research), partner site Munich Heart Alliance, 80802 Munich, Germany

Address of correspondence:

Stefan Coassin, PhD
Division of Genetic Epidemiology
Department of Medical Genetics, Molecular and Clinical Pharmacology
Medical University of Innsbruck
Schöpfstr. 41, A-6020 Innsbruck, AUSTRIA
Phone: (+43) 512 9003 70576, Fax: (+43) 512 9003 73561
E-mail: Stefan.Coassin@i-med.ac.at

Contents

Contents 2
Supplementary Note 3
Supplementary Figures 4
Supplementary Figure I: 4
Supplementary Figure II: 5
Supplementary Figure III: 6
Supplementary Figure IV: 7
Supplementary Figure V: 8
Supplementary Figure VI: 9
Supplementary Figure VII: 10
Supplementary Figure VIII: 11
Supplementary Tables 12
Supplementary Table I: 12
Supplementary Table II: 12
Supplementary Table III: 13
Supplementary Table IV: 14
Supplementary Table V: 15
Supplementary Table VI: 16
Supplementary Table VII: 17
Supplementary Table VIII: 18
References 18

Supplementary Note

The study was supported by the Austrian Genome Project 'GOLD' to FK, by the Austrian Science Fund (FWF) Project Number P266600-B13 to CL and by the intramural funding of the Medical University of Innsbruck for young scientists (MUI-START, project 2015-06-007) to SC.

The Young Finns Study has been financially supported by the Academy of Finland: grants 286284 (T.L.), 134309 (Eye), 126925, 121584, 124282, 129378 (Salve), 117787 (Gendi), and 41071 (Skidi); the Social Insurance Institution of Finland; Kuopio, Tampere and Turku University Hospital Medical Funds (grant X51001 for T.L.); Juho Vainio Foundation; Paavo Nurmi Foundation; Finnish Foundation of Cardiovascular Research (T.L.); Finnish Cultural Foundation; Tampere Tuberculosis Foundation (T.L.); Emil Aaltonen Foundation (T.L.); Yrjö Jahnsson Foundation (T.L.). and Signe ja Ane Gyllenberg's Foundation (T.L). and Diabetes Research Foundation of Finnish Diabetes Association (T.L). We gratefully acknowledge the THL DNA laboratory for its skillful work to produce the DNA samples used in this study and Irina Lisinen for the expert technical assistance in the statistical analyses.

The CoLaus study was and is supported by research grants from GlaxoSmithKline, the Faculty of Biology and Medicine of Lausanne, and the Swiss National Science Foundation (grants 33CSCO-122661, 33CS30-139468 and 33CS30-148401).

The KORA-Study Group consists of A. Peters (speaker), J. Heinrich, R. Holle, R. Leidl, C. Meisinger, K. Strauch, and their co-workers, who are responsible for the design and conduct of the KORA studies. The KORA study was initiated and financed by the Helmholtz Zentrum München – German Research Center for Environmental Health, which is funded by the German Federal Ministry of Education and Research (BMBF) and by the State of Bavaria. Furthermore, KORA research was supported within the Munich Center of Health Sciences (MC-Health), Ludwig-Maximilians-Universität, as part of LMUinnovativ.

Supplementary Figures

Supplementary Figure I:

Full boxplots of Lp(a) concentrations for each population.

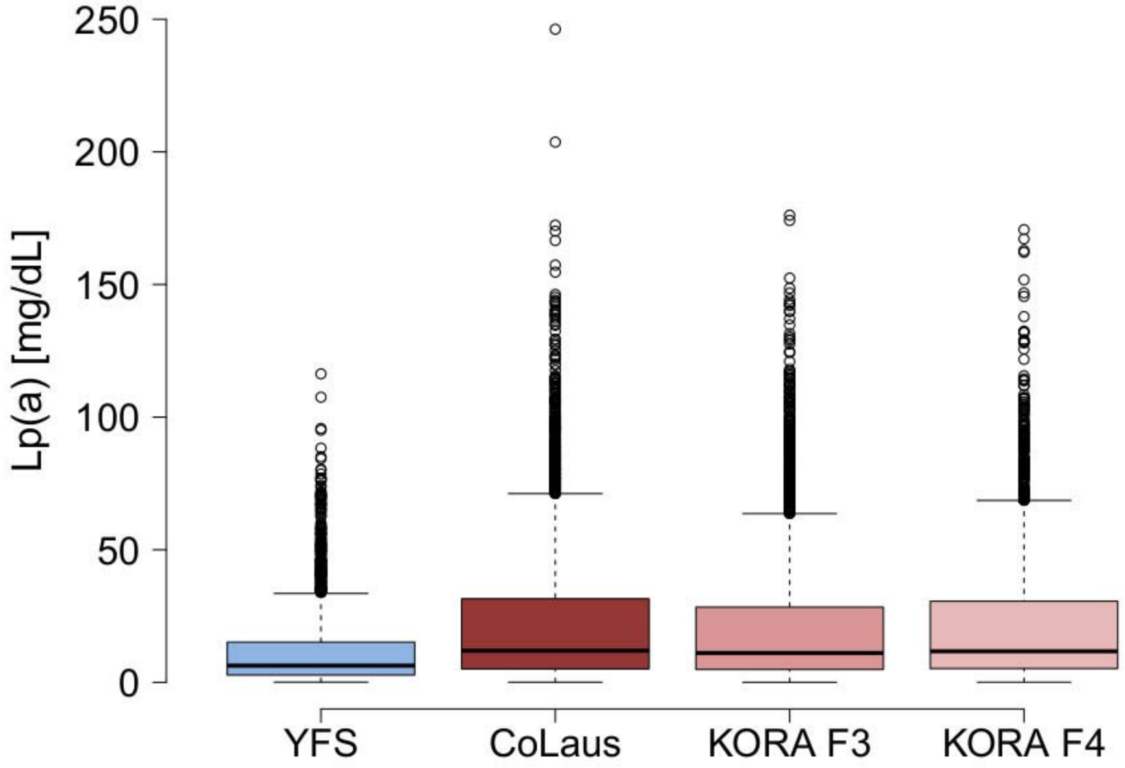

Supplementary Figure II:

Distribution of Lp(a) concentrations

Frequencies of Lp(a) concentrations in studies. Percent of individuals of each study in the bins of 5 mg/dL Lp(a) are shown. An elevated proportion of YFS shows Lp(a) concentrations of 0-5 mg/dL.

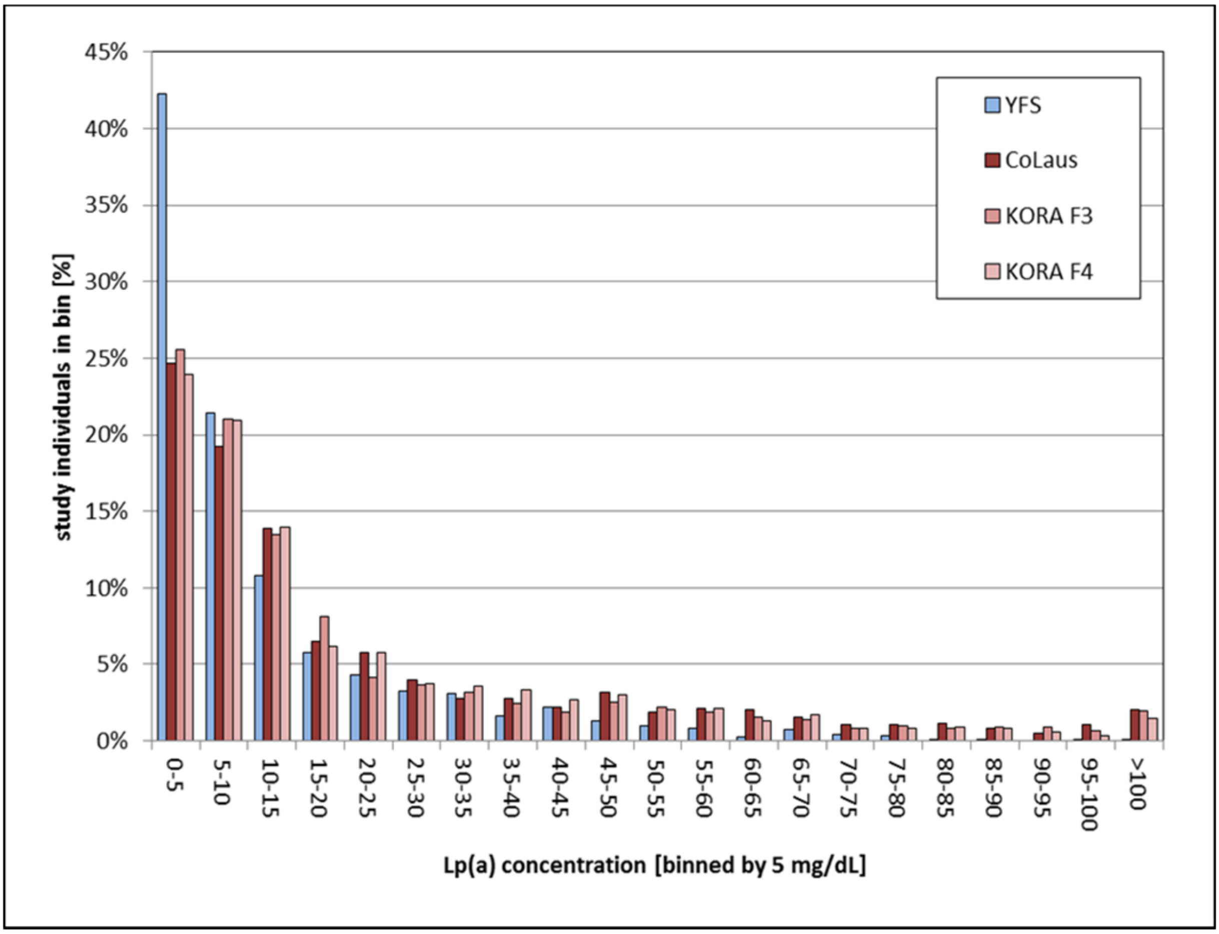

Supplementary Figure III:

Percentage of populations with Lp(a) concentrations >30 mg/dL and >50 mg/dL

Percentage of YFS and NFE individuals presenting Lp(a) concentrations increasing CVD risk (>30[1] and >50[2] mg/dL).

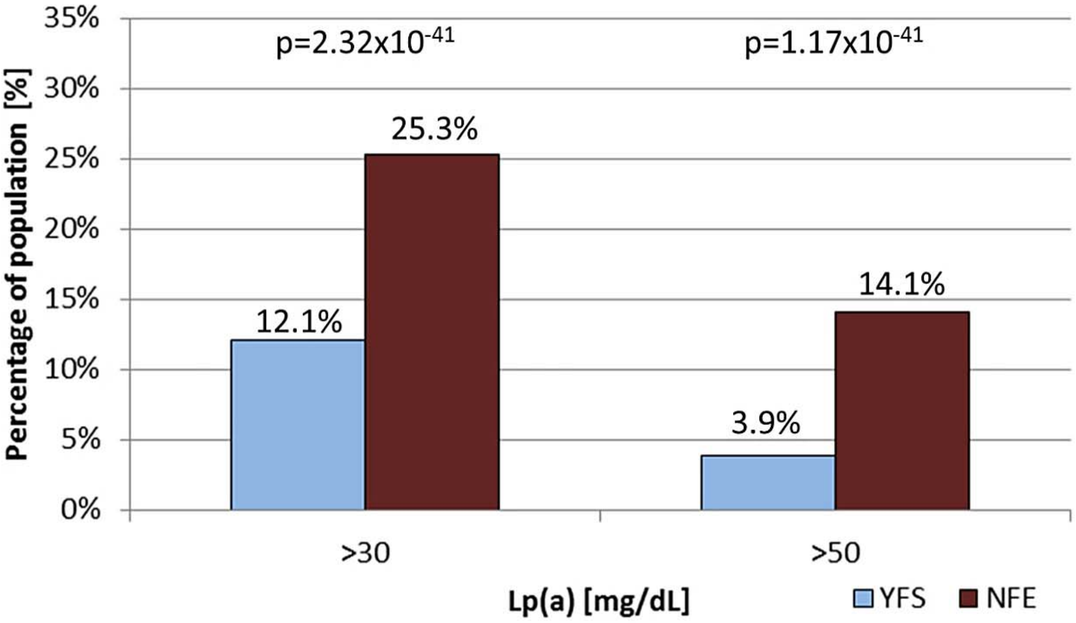

Supplementary Figure IV:

Apo(a) isoform distribution in the populations.

A: Apo(a) isoform 1 distribution in the populations. Panel A shows the apo(a) isoform 1 distribution in the different populations. YFS has markedly higher frequencies of carriers of isoforms containing >28 KIV repeats in isoform 1, so subsequently in YFS is also a higher frequency of probands grouped into HMW, which is shown in panel B. Table with the numbers of probands in each group is given in Figure 3 of the main manuscript.

B: Frequencies LMW vs. HMW. Frequencies of probands in each study grouped into LMW or HMW are shown. YFS show reduced LMW frequency and accordingly a higher HMW frequency compared to the NFE-populations.

C and D: Apo(a) isoform distribution of the two alleles. The Violin plots show distributions of the smaller allele (Isoform 1, panel C) and the larger allele (if detectable on the Western blot, Isoform 2, panel D) in the populations. In both panels the distribution of the apo(a) isoforms is shifted towards larger alleles in YFS compared to the other populations. In the Isoform 2, the samples in the CoLaus study have the same median apo(a) isoform as YFS.

diamonds: median; black dots: 25th, 75th percentile; red dots: 10th and 90th percentiles

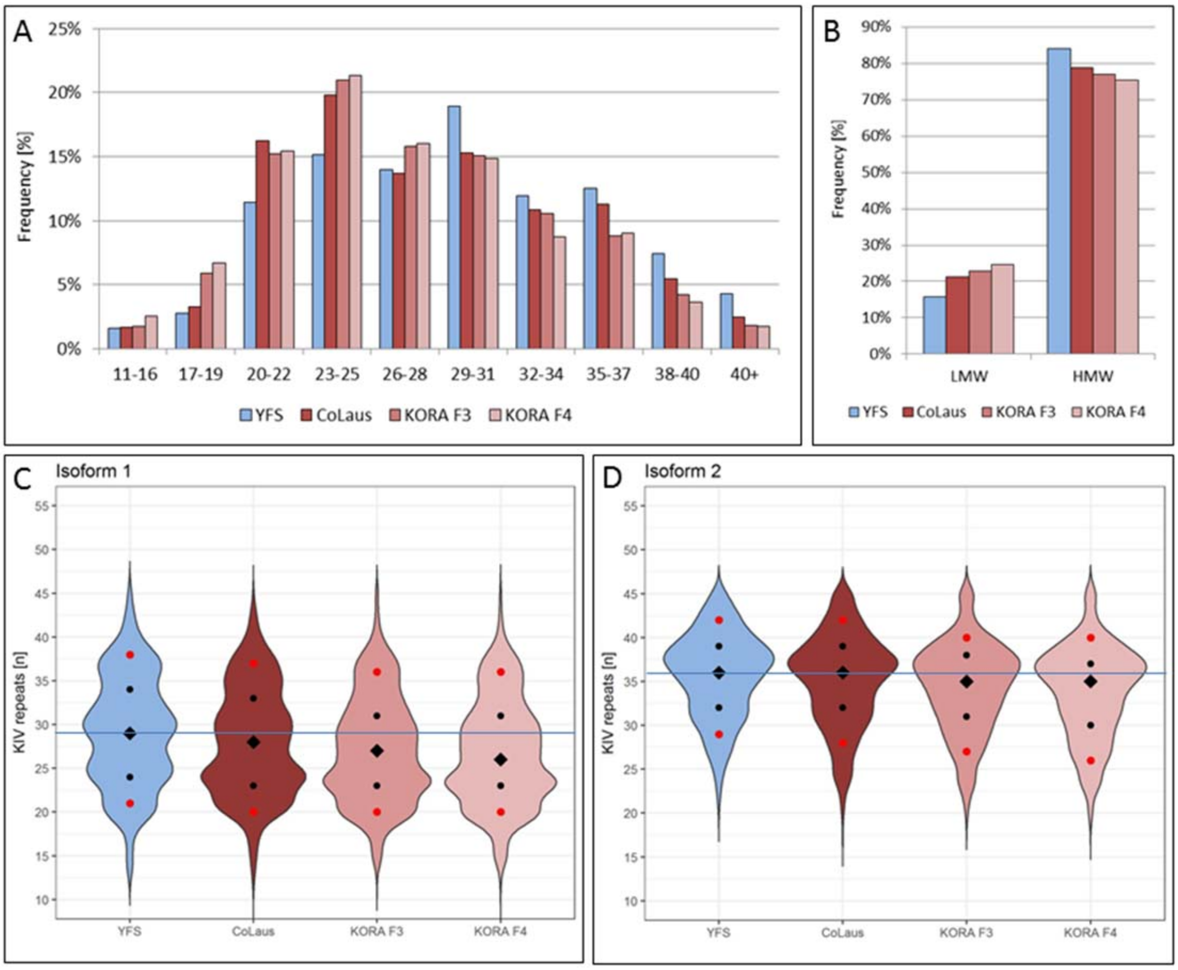

Supplementary Figure V:

Boxplots of Lp(a) concentrations by LMW/HMW status.

A: Lp(a) in LMW carriers. The boxplots of Lp(a) concentrations are shown for all LMW isoform carriers in each population.

B: Lp(a) in HMW carriers. The boxplots of Lp(a) concentrations are shown for all HMW isoform carriers in each population.

The exact median and quartile values are reported in Table 1 of the main document.

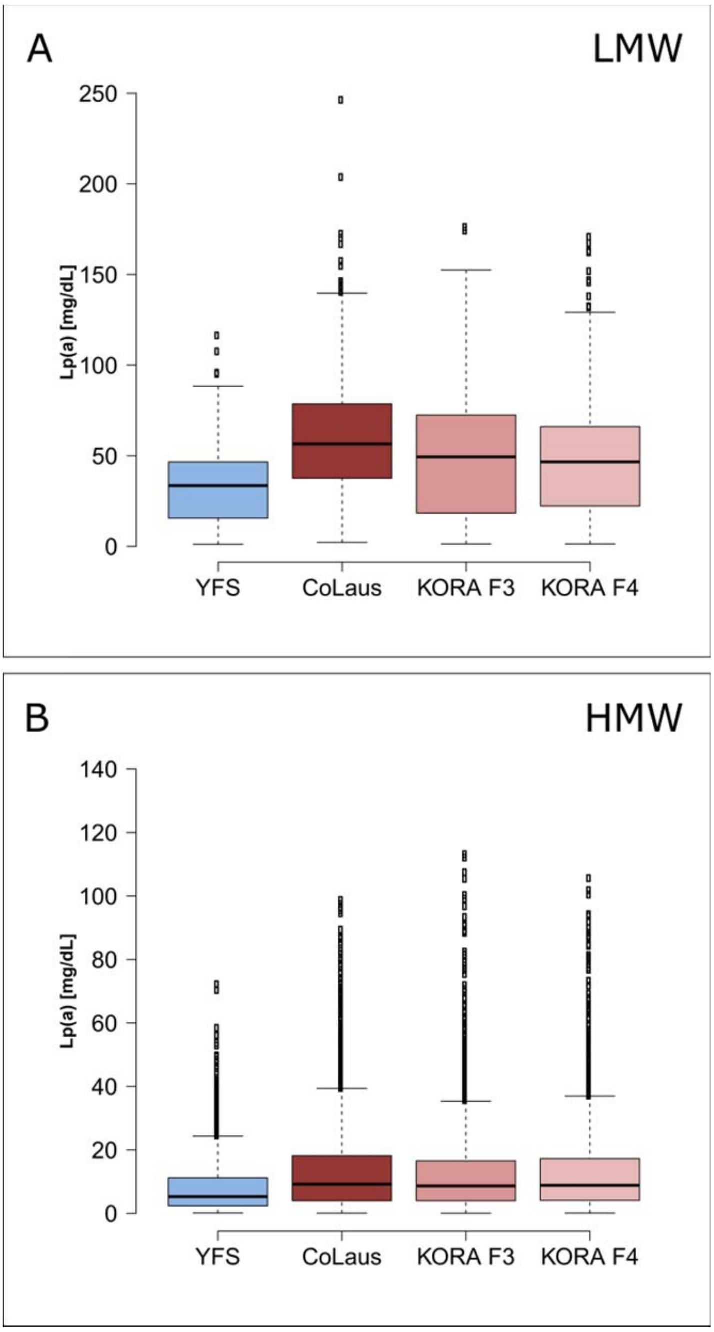

Supplementary Figure VI:

Median Lp(a) concentrations.

Histogram showing the median Lp(a) concentration in each population stratified by KIV repeats. YFS have lower Lp(a) concentrations than the NFE subgroups over the entire isoform range. The number of probands in each group is given in Supplementary Table IV and the exact values are given in Supplementary Table V.

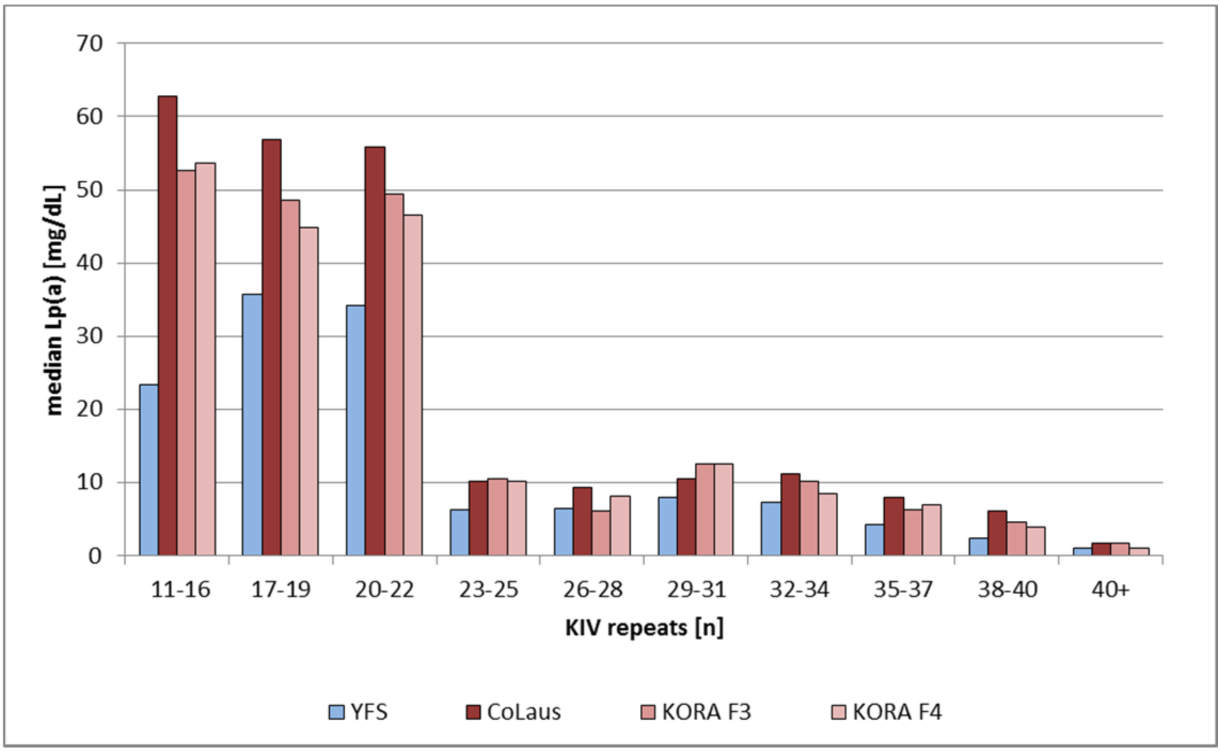

Supplementary Figure VII:

Histograms showing the distribution of rs10455872 (A) and rs3798220 (B) in the different populations. No differences in the distribution were observed (p=0.20 and p=0.61).The

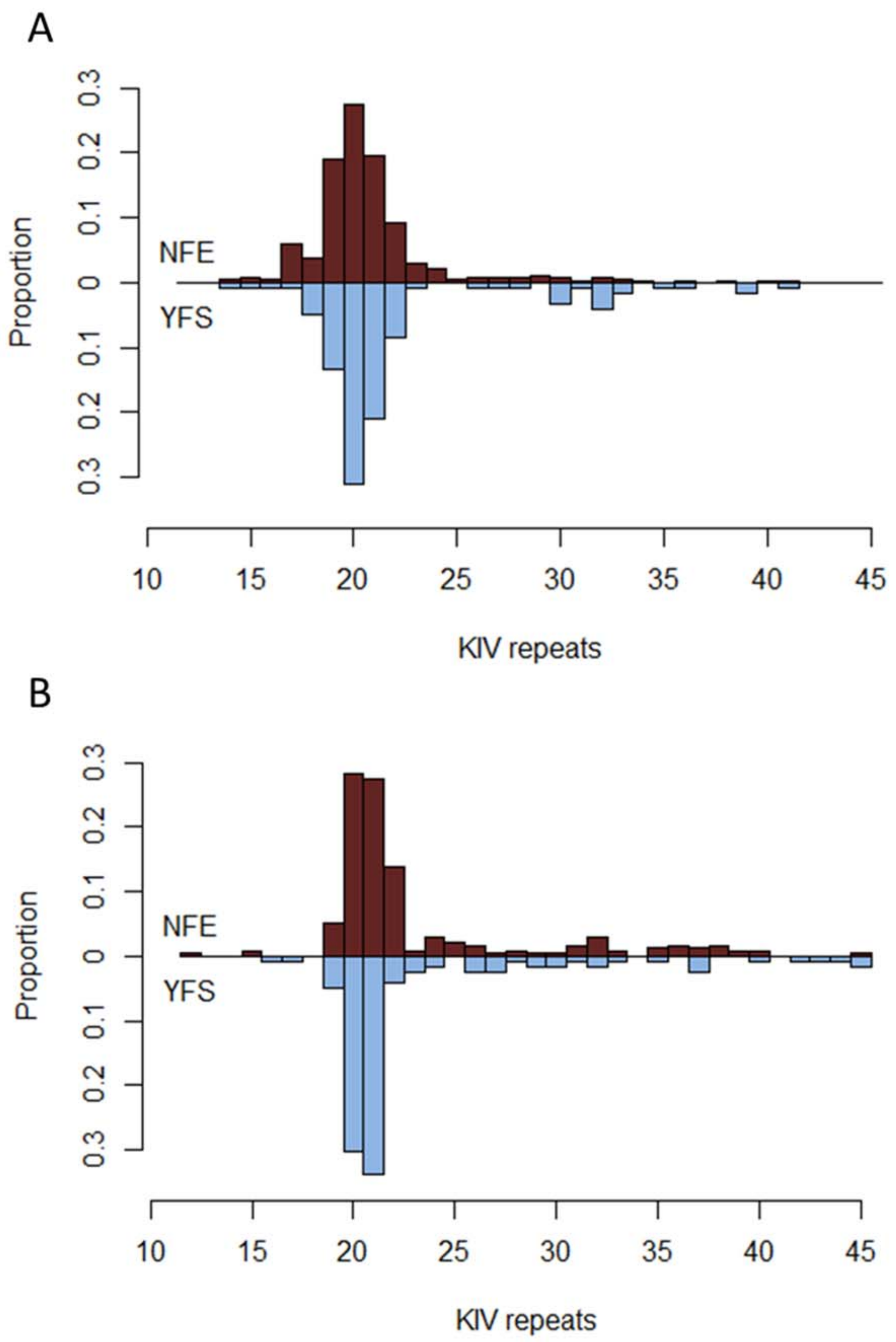

Supplementary Figure VIII:

Nonlinear Spline

Spline showing the effect of the small isoform (isoform 1) on inverse normal transformed Lp(a) levels. The spline are centered at 0 at isoform 1 = 27 (median on the NFE studies) and is derived from the multiple regression model additionally adjusting for the variables age, sex, eGFR, rs143411368, rs41272114, rs11591147, rs10455872, rs3798220, APOE2 and APOE4-carriers (Supplementary Table VI). se: standard error.

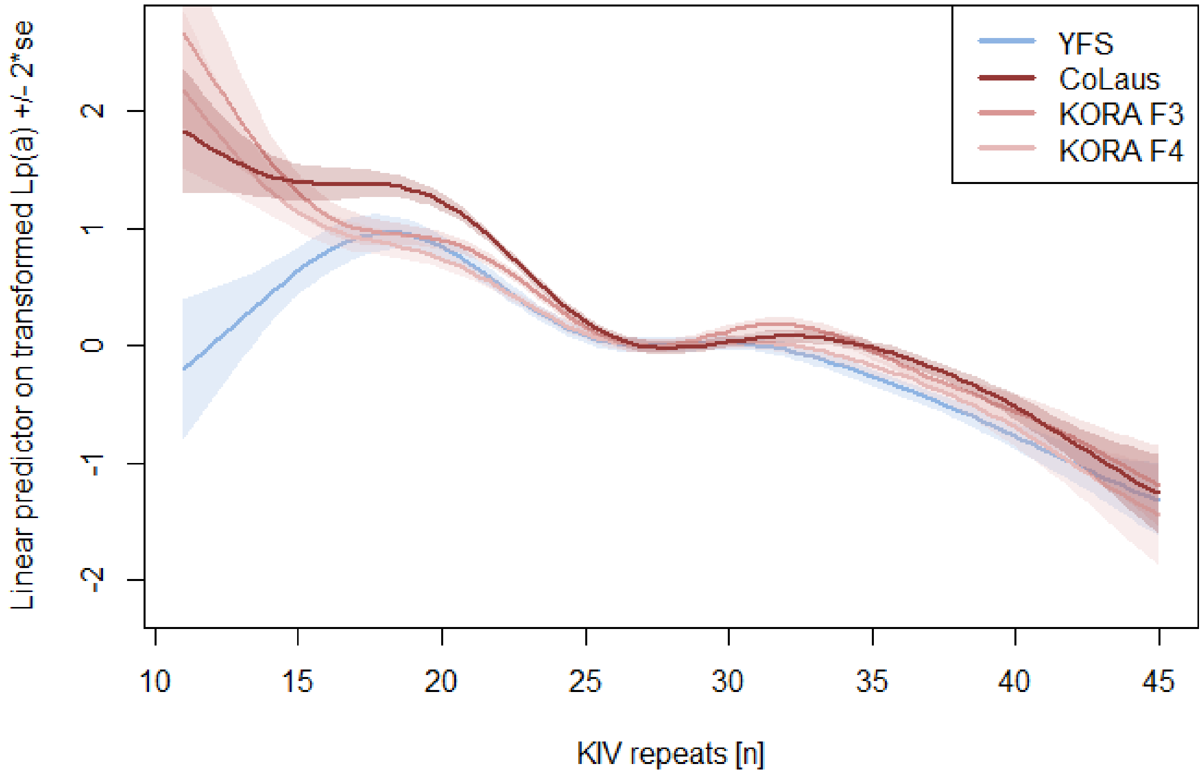

Supplementary Tables

Supplementary Table I:

Lp(a) concentrations [mg/dL] stratified by (a) sex and (b) additionally including only participants in the age range ≥32 to ≤39 years, where all studies overlap.

Study	25th percentile	Median	Mean	75th percentile	Wilcoxon-test p-value
**All participants**					
**Men, YFS (n= 1,026)**	2.62	6.09	12.15	14.39	9.66×10^{-36}
**Men, NFE (n= 4,773)**	4.46	10.65	20.98	28.16	
**Women, YFS (n= 1,255)**	2.86	6.56	12.70	15.74	5.61×10^{-59}
**Women, NFE (n= 5,230)**	5.62	12.64	23.83	32.51	
**Age range ≥32 to ≤39 years.**					
**Men, YFS (n=528)**	2.75	6.42	12.79	15.19	3.083×10^{-05}
**Men, NFE (n=311)**	4.01	9.58	17.96	21.27	
**Women, YFS (n=658)**	2.80	6.58	12.67	15.59	1.42×10^{-07}
**Women, NFE (n=354)**	4.80	10.04	17.89	22.94	

Supplementary Table II:

Lp(a) concentrations in percentiles.

Lp(a) concentration percentile data is shown in YFS and NFE - both grouped and split up in single populations.

	Percentiles Lp(a) [mg/dL]						
**Study**	**5**	**10**	**25**	**50**	**75**	**90**	**95**
**YFS (n=2,281)**	0.96	1.33	2.78	6.32	15.19	33.05	45.7
**NFE (n=10,003)**	1.57	2.29	5.07	11.59	30.43	60.35	79.84
CoLaus (n=3,998)	1.42	2.17	5.1	11.94	31.51	62.74	81.71
KORA F3 (n=3,089)	1.82	2.52	4.89	11.07	28.4	59.48	81.26
KORA F4 (n=2,916)	1.46	2.13	5.23	11.73	30.6	56.36	73.19

Supplementary Table III:

Apo(a) isoform percentiles of both isoforms

Table shows percentiles of the apo(a) isoform size stratified in the smaller and – if determinable – the larger isoform in YFS and NFE, both grouped and in single populations. In YFS both isoforms are larger than in NFE.

		Percentiles KIV repeats						
**Isoform**	**Study**	**5**	**10**	**25**	**50**	**75**	**90**	**95**
**Isoform 1**	**YFS (n=2,281)**	20.0	21.0	24.0	29.0	34.0	38.0	40.0
	**NFE (n=10,003)**	19.0	20.0	23.0	27.0	32.0	36.0	38.0
	CoLaus (n=3,998)	20.0	20.0	23.0	28.0	33.0	37.0	39.0
	KORA F3 (n=3,089)	19.0	20.0	23.0	27.0	32.0	36.0	38.0
	KORA F4 (n=2,916)	19.0	20.0	23.0	26.0	31.0	36.0	38.0
**Isoform 2**	**YFS (n=2,281)**	27.0	29.0	32.0	36.0	39.0	42.0	43.0
	**NFE (n=10,003)**	25.0	27.0	31.0	35.0	38.0	40.0	42.0
	CoLaus (n=3,998)	25.0	28.0	32.0	36.0	39.0	42.0	43.0
	KORA F3 (n=3,089)	25.0	27.0	31.0	35.0	38.0	40.0	42.0
	KORA F4 (n=2,916)	24.0	26.0	30.0	35.0	37.0	40.0	41.0

Supplementary Table IV:

Numbers of probands in isoform 1 strata of all studies

	Probands in each group [n]									
**KIV-repeats (grouped)**	**11-16**	**17-19**	**20-22**	**23-25**	**26-28**	**29-31**	**32-34**	**35-37**	**38-40**	**40+**
**YFS (n=2,281)**	37	63	261	345	319	431	273	286	169	97
**NFE (n=10,003)**	194	510	1568	2061	1502	1510	1013	985	455	205
CoLaus	66	131	649	793	548	610	433	450	218	100
KORA F3	54	183	470	647	487	466	325	272	130	55
KORA F4	74	196	449	621	467	434	255	263	107	50

Supplementary Table V:

Median [25%/75% percentiles] Lp(a) concentrations per isoform group

Table shows the median and the 25%/75% percentiles for each apo(a) isoform group.

	Isoform group									
**Lp(a) [mg/dL]**	**11-16**	**17-19**	**20-22**	**23-25**	**26-28**	**29-31**	**32-34**	**35-37**	**38-40**	**40+**
**YFS (n=2,281)**	23.41 [4.45 / 39.80]	35.79 [15.40 / 45.58]	34.15 [20.22 / 50.68]	6.32 [4.18 / 14.08]	6.53 [2.35 / 16.08]	8.02 [2.89 / 14.05]	7.24 [3.59 / 11.10]	4.21 [2.29 / 6.55]	2.49 [1.58 / 4.46]	1.05 [0.69 / 1.83]
**NFE (n=10,003)**	56.43 [42.17 / 78.23]	48.54 [27.89 / 67.57]	51.23 [25.72 / 73.09]	10.33 [6.30 / 22.68]	8.19 [2.97 / 22.03]	11.62 [3.92 / 20.85]	10.26 [5.30 / 15.96]	7.05 [4.00 / 11.71]	4.91 [2.71 / 8.27]	1.44 [0.71 / 2.82]
CoLaus (n=3,998)	62.76 [48.54 / 88.28]	56.94 [40.93 / 73.15]	55.86 [35.07 / 78.53]	10.20 [6.38 / 23.30]	9.39 [3.17 / 23.56]	10.59 [3.28 / 21.37]	11.27 [5.27 / 18.18]	7.94 [4.19 / 12.61]	6.08 [2.98 / 9.61]	1.68 [0.85 / 3.39]
KORA F3 (n=3,089)	52.62 [41.14 / 70.87]	48.55 [16.42 / 68.00]	49.35 [18.28 / 73.97]	10.50 [6.46 / 22.07]	6.08 [2.94 / 19.62]	12.64 [4.49 / 20.11]	10.19 [5.60 / 15.53]	6.24 [3.78 / 9.70]	4.64 [2.74 / 7.40]	1.75 [0.73 / 2.28]
KORA F4 (n=2,916)	53.66 [34.59 / 70.04]	44.87 [30.76 / 60.85]	46.59 [16.21 / 66.09]	10.16 [6.20 / 23.03]	8.21 [2.79 / 21.88]	12.49 [4.62 / 20.26]	8.54 [4.65 / 14.35]	7.00 [3.95 / 11.69]	4.01 [2.34 / 7.11]	1.00 [0.59 / 1.81]

Supplementary Table VI:

Results of multiple regression analysis in all studies individually. The effect of the isoforms (isoform 1) on Lp(a) and inverse normally transformed Lp(a) levels in all individual studies is shown as nonlinear spline in Figure 5 in the main document.

Variable	Results from multiple regression model on Lp(a)* in							
	YFS		KORA F3		KORA F4		CoLaus	
	β	p	β	p	β	p	β	p
Age	0.07	0.3962	0.13	0.0025	0.1	0.0002	0.14	$9.26x10^{-06}$
Sex (f)	0.62	0.0368	2.72	$2.21x10^{-09}$	2.46	$2.61x10^{-05}$	2.84	$5.40x10^{-09}$
Isoforms$	--	$2.60x10^{-124}$	--	$1.19x10^{-165}$	--	$1.51x10^{-132}$	--	$1.10x10^{-242}$
eGFR	-0.03	0.1899	-0.04	0.0073	-0.03	0.2565	-0.02	0.4521
rs143411368#	-2.6	$5.33x10^{-09}$	-3.57	0.3511	-8.19	0.0093	1.29	0.5796
rs41272114#	-2.45	$6.38x10^{-19}$	-4.4	$2.28x10^{-14}$	-4.66	$5.69x10^{-14}$	-6.55	$4.17x10^{-32}$
rs11591147#	0.53	0.9545	-4.11	0.0056	0.66	0.5601	1.87	0.5118
rs10455872#	11.68	$1.33x10^{-13}$	24.12	$6.68x10^{-39}$	20.98	$1.17x10^{-28}$	13.14	$8.59x10^{-17}$
rs3798220#	28.03	$3.37x10^{-14}$	41.13	$2.42x10^{-26}$	43.83	$4.44x10^{-31}$	44.3	$6.95x10^{-35}$
APOE2 carrier	-2.34	0.0071	-5.02	$3.80x10^{-11}$	-2.19	0.0026	-3.01	$8.44x10^{-06}$
APOE4 carrier	-0.21	0.6147	-1.96	0.0100	0.48	0.4403	0.06	0.8923

*β estimates are derived from models on Lp(a); p-values are derived from models on inverse normal transformed Lp(a) values to ensure normal distribution assumption

$ p-values for isoforms refers to the significance of a nonlinear spline of isoform 1 on inverse-normal transformed Lp(a); for effect size: see nonlinear spline in Figure 4 and Supplementary Figure VIII

For SNPs, an additive inheritance model was assumed

Supplementary Table VII:

Mediation analysis results on inverse normal transformed Lp(a) including only those variables, which qualify for mediation analysis

Variable	YFS		Identified as mediator	Relative effects	
	P-value 1	P-value 2		estimate §	95 % CI*
**Explained effects†**				0.718	[0.634, 0.806]
Age	<0.001	<0.001	✓	0.264	[0.185, 0.348]
Sex (f)	<0.001	0.019	✓	-0.006	[-0.013, -0.001]
Apo(a) isoforms	<0.001	<0.001	✓	0.273	[0.235, 0.310]
eGFR	<0.001	<0.001	✓	0.104	[0.052, 0.157]
rs143411368	<0.001	<0.001	✓	0.038	[0.024, 0.055]
rs41272114	<0.001	<0.001	✓	0.040	[0.024, 0.054]
rs11591147	0.601	--	--	--	--
rs10455872	<0.001	0.020	✓	0.025	[0.010, 0.039]
rs3798220	<0.001	0.164	--	--	--
APOE2 carrier	<0.001	<0.001	✓	-0.018	[-0.025, -0.011]
APOE4 carrier	0.103	<0.001	--	--	--
**Unexplained effect ‡**				0.282	[0.194,0.366]

P-value 1: Type-3 tests in the full model, testing, whether factors are associated with Lp(a); P-value 2: Tests of relationship of each factor with the predictor (YFS/NFE-difference)
*95%CI were derived with bootstrap samples assuming normal approximation
† "indirect effect" of the mediation analysis
‡ "direct effect" of the mediation analysis
§ estimates rounded, may not add up to exactly to 1

Supplementary Table VIII:

Imputation details

This table shows the imputation details and quality of the populations in this study.

	YFS	CoLaus	KORA F3	KORA F4
**Microarray used**	Illumina 670k	Affymetrix GeneChip® Human Mapping 500K	Illumina Omni 2.5/ Illumina Omni Express	Affymetrix Axiom
**QC before imputation**	callrate >95%, p-value HWE > 10^{-6}, MAF>1%	callrate >90%*, p-value HWE > 10^{-7}	callrate >98%, p-value HWE > 10^{-10}	callrate >98%, p-value HWE > 10^{-10}
**Program used for imputation**	IMPUTE	minimac	IMPUTE	IMPUTE
**Reference panel for imputation**	1000G phase1v3[3]	1000G phase1v3[3]	HRC panel[4]	HRC panel[4]

References

1. Kamstrup PR, Tybjaerg-Hansen A, Steffensen R, Nordestgaard BG. Genetically elevated lipoprotein(a) and increased risk of myocardial infarction. *JAMA*. 2009;301:2331-2339.

2. Nordestgaard BG, Chapman MJ, Ray K, et al. Lipoprotein(a) as a cardiovascular risk factor: current status. *Eur Heart J*. 2010;31:2844-2853.

3. Auton A, Abecasis GR, Altshuler DM, et al. A global reference for human genetic variation. *Nature*. 2015;526:68-74.

4. Loh P-R, Danecek P, Palamara PF, et al. Reference-based phasing using the Haplotype Reference Consortium panel. *Nat Genet*. 2016;48:1443-1448.
