# Supplementary material for: Genetic Factors Explain a Major Fraction of the 50% Lower Lipoprotein(a) Concentrations in Finns
Source: Arterioscler Thromb Vasc Biol. 2018 Mar 22;38(5):1230–41. doi: 10.1161/ATVBAHA.118.310865 (PMC5943067; doi:10.1161/ATVBAHA.118.310865)

Factors explaining the 50% lower Lp(a) levels in a Finnish population compared to Central Europeans

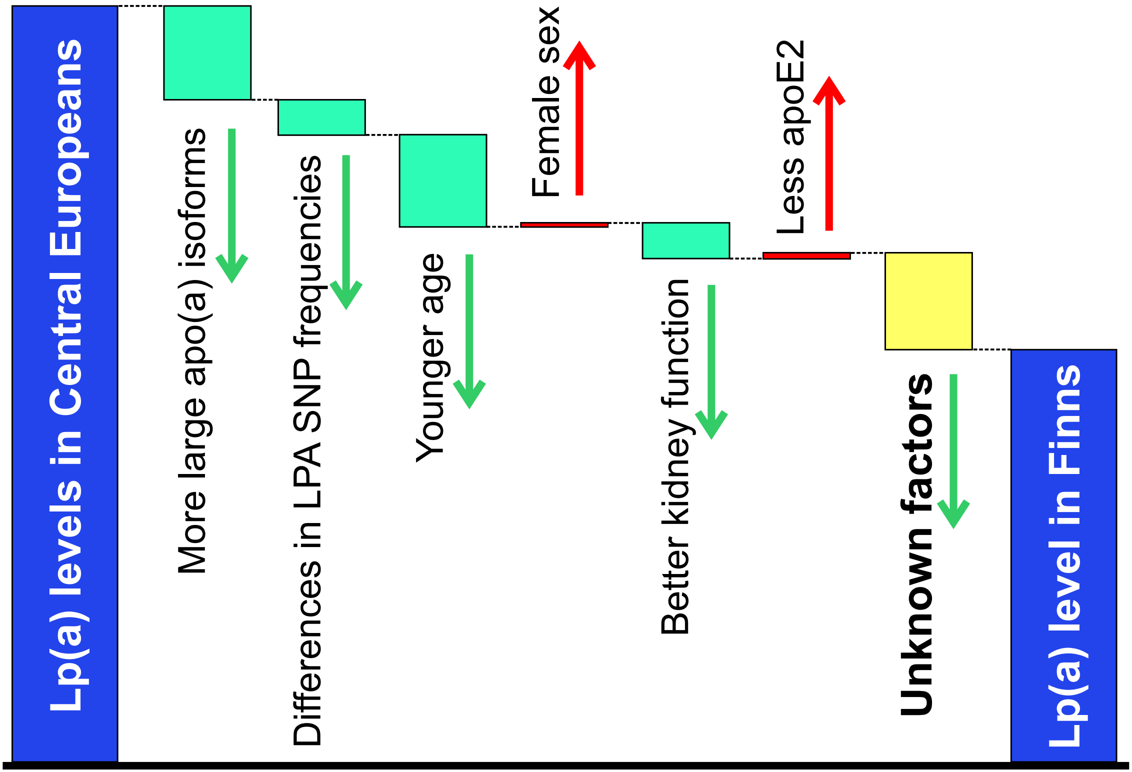

Supplement: Supplementary file 3 [file atv-38-1230-s003.pdf]
